# Exploring the Dietary Patterns and Health Behaviours of Centenarians in Ourense (Spain): Adherence to the Southern European Atlantic Diet

**DOI:** 10.3390/nu17132231

**Published:** 2025-07-05

**Authors:** Pablo García-Vivanco, Roberto Fernandez, Rosa Meijide-Faílde, Esperanza Navarro-Pardo, Cristina Conde, Ricardo de la Fuente, Cristina Margusinos, Alberto Rodríguez, Ana Canelada, Pablo Taboada, Alberto Cepeda, Alberto Coelho

**Affiliations:** 1 Spanish Academy of Nutrition and Dietetics, 31006 Pamplona, Spain; 2 Nutrition and Digestive Working Group, Spanish Society of Clinical, Family, and Community Pharmacy (SEFAC), 28045 Madrid, Spain; 3 Allariz Health Center, Galician Health Service (SERGAS), San Rosendo Foundation, 32660 Ourense, Spain; 4 Cell Therapy and Regenerative Medicine Research Group, Institute of Biomedical Research of La Coruña (INIBIC), Interdisciplinary Centre for Chemistry and Biology (CICA), University of A Coruña, 15071 A Coruña, Spain; 5 Department of Physiotherapy, Medicine and Biomedical Sciences, University of A Coruña, 15071 A Coruña, Spain; 6 Department of Developmental and Educational Psychology, University of Valencia, 46010 Valencia, Spain; 7 Official College of Pharmacists of Ourense, 32004 Ourense, Spain; 8 Official College of Dietitians-Nutritionists of Galicia, 15190 A Coruña, Spain; 9 Toén Health Center, Galician Health Service (SERGAS), 32930 Toén, Spain; 10 Verín Health Center, Galician Health Service (SERGAS), 32600 Verín, Spain; 11 Department of Health Sciences, University of Málaga, 29016 Málaga, Spain; 12 Institute of Materials-USC (IMATUS), University of Santiago de Compostela, 15782 Santiago de Compostela, Spain; 13 Colloids and Polymers Physics Group, Department of Physics of Particles, Faculty of Physics, University of Santiago de Compostela, 15782 Santiago de Compostela, Spain; 14 Department of Analytical Chemistry, Nutrition and Food Science, University of Santiago de Compostela, 15782 Santiago de Compostela, Spain; 15 Department of Organic Chemistry, Faculty of Pharmacy, University of Santiago de Compostela, 15782 Santiago de Compostela, Spain

**Keywords:** longevity, epigenetics, centenarians, southern European Atlantic diet

## Abstract

**Background:** Understanding the multifactorial determinants of human longevity remains a major scientific challenge. Certain regions of the world—so-called “longevity hotspots”—exhibit a notably high prevalence of centenarians; one such region is the province of Ourense, in north-western Spain. **Objectives:** This study aimed to analyse, for the first time, the nutritional factors associated with healthy longevity among centenarians, as well as those linked to longevity irrespective of health status, in the province of Ourense. **Methods:** A cross-sectional, retrospective, observational, mixed-methods study was conducted. A population of 261 individuals aged 100 or over residing in Ourense was identified. A sample of 156 participants was included in the quantitative analysis; from this sample, 25 centenarians were selected for in-depth qualitative analysis through personal interviews. **Results:** Dietary patterns aligned with the Southern European Atlantic Diet (SEAD), combined with strong social bonds and a culture of self-sufficiency, appear to be key contributors to exceptional longevity in this population. **Conclusions:** Remarkable longevity in Ourense is associated with a combination of factors: adherence to an SEAD-style dietary pattern, an active and uncomplicated lifestyle, and strong social support networks.

## 1. Introduction

Fighting for longevity is neither a recent human aspiration nor a novel scientific endeavour. The first article on this topic was published in 1838, at a time when the average human life expectancy was approximately 40 years [1].

The classification of long-lived individuals into three phenotypic subgroups—escapers, delayers, and survivors—was first introduced by Perls and colleagues within the framework of the New England Centenarian Study (NECS), one of the most comprehensive longitudinal investigations on human longevity [2]. Although not a formal clinical classification, it has become a widely adopted conceptual tool in the field of gerontology, aiding the analysis of heterogeneity among centenarians and providing insight into how genetic, environmental, and lifestyle factors contribute to healthy ageing.

In this model, *escapers* are individuals who reach extreme old age—typically 100 years or more—without developing major chronic illnesses. *Delayers* are those who postpone the onset of such conditions until after the age of 85. *Survivors* develop chronic illnesses before the age of 85 yet still reach 100 years [3].

There is more uncertainty than certainty regarding the determinants of longevity. Genetics plays an important, though variable, role in extreme longevity; however, its influence has often been overestimated. A study on Scandinavian twins concluded that genetic inheritance accounts for only 20% to 30% of life expectancy, while environmental influences represent at least 70% [4]. Heritability has been estimated to range between 15% and 40%, but at advanced ages—such as those reached by centenarians, who have surpassed the most common causes of death—genetic factors appear to outweigh environmental ones. The genes associated with longevity influence mitochondrial function, resistance to oxidative stress, metabolism, DNA repair, cell cycle regulation, proteostasis, telomere shortening, and other targets involved in the ageing process.

Epigenetics is increasingly recognised as a key aspect of ageing and longevity, as it involves heritable changes not in the DNA sequence itself but in how the DNA is packaged, which can be shaped by environmental factors and transmitted to subsequent generations [5]. One such process is DNA methylation. Centenarians appear to delay age-related changes in DNA methylation and may pass on this ability to their descendants [6]. It appears that centenarians and supercentenarians (individuals who have reached the age of 110 or above, regardless of health status) exhibit epigenetic profiles that are younger than expected [7]. So-called “epigenetic diets” have been described [8], which may favourably influence an individual’s epigenetic profile. These diets include natural compounds capable of mediating such effects, such as resveratrol, spermidine, metformin, and selenium, and dietary components like green tea, broccoli sprouts, and soy [9]. However, existing data on centenarians’ nutritional habits and caloric intake are neither sufficiently reliable nor detailed. Antioxidants, for instance, have long attracted attention as potentially preventive agents. Nevertheless, current evidence does not suggest that ascorbate (vitamin C), tocopherol (vitamin E), lycopene, selenium, or beta-carotene have a protective effect on life expectancy, although there is some evidence suggesting that retinol has a potential benefit [10]. Similar uncertainty surrounds omega-3 fatty acids and sulforaphane found in the diet [11,12].

In the context of nutrition, research has shown that certain foods can influence epigenetic markers. Some vitamins may affect DNA methylation patterns and histone modifications, potentially impacting gene expression and disease risk. Thus, dietary interventions may contribute to epigenetic regulation, highlighting the role of nutrition in maintaining health and preventing disease through epigenetic mechanisms. MicroRNAs (miRNAs) regulate the metabolism of omega-3 and omega-6 fatty acids (FAs), which is important for inflammation, lipid metabolism, and insulin sensitivity. Fish and seed oils are sources of omega-3 polyunsaturated fatty acids (PUFAs), which can downregulate pro-inflammatory miRNAs, thereby reducing chronic inflammation, cardiovascular diseases (CVDs), and related conditions [13].

Omega-3 fatty acids reduce inflammation, improve lipid profiles, and enhance insulin sensitivity. In contrast, diets high in omega-6 polyunsaturated fatty acids (PUFAs) increase the expression of pro-inflammatory microRNAs (miRNAs), which are associated with chronic inflammation, cancer, and cardiovascular disease (CVD). A balanced omega-3 to omega-6 ratio is therefore essential for regulating miRNA expression and mitigating the risk of disease [14,15].

Studies have shown that food intake can modulate miRNA levels, presenting a potential therapeutic strategy. Omega-3 PUFAs derived from fish oil downregulate miRNAs linked to inflammation and lipid metabolism, thereby reducing the risk of CVD. They also affect miR-33, a regulator of cholesterol homeostasis, contributing to improved lipid profiles and reduced atherosclerosis [16]. In addition to omega-3s, natural polyphenols such as resveratrol and quercetin—found in apples, onions, and tea—exert cardioprotective effects through miRNA modulation. Resveratrol upregulates miRNAs that enhance endothelial function and reduce inflammation, while quercetin regulates miRNAs involved in vascular smooth muscle cell function, preventing arterial stiffness and hypertension.

The maintenance of a healthy gut microbiota has been associated with longevity and centenarian status, including among supercentenarians (individuals aged 110 years or more). A healthy microbiome may help counteract systemic inflammation, maintain intestinal barrier integrity, and preserve bone and cognitive function [17].

With regard to lifestyle and psychological factors, both sleep quality [18] and duration have been linked to longevity, in part through their influence on telomere length [19]. Socioeconomic status is another important determinant of longevity, including both current and perceived socioeconomic status [20] and socioeconomic conditions during early life [21]. Spirituality is frequently cited as a factor associated with a longer life expectancy; however, further studies are needed to clarify whether religiosity and spirituality contribute directly to longer and healthier lifespans [22]. More consistent evidence exists concerning the association between longevity and certain personality traits, particularly a positive outlook and strong adaptability in the face of adversity. These traits can be classified according to the Big Five personality dimensions: extraversion, agreeableness, conscientiousness, openness to experience, and emotional stability [23].

Environmental influences on longevity have also been investigated. Variables such as elevation, climate, and the presence of UNESCO World Heritage sites have been identified as potential contributing factors [24]. Some Chinese populations with notable concentrations of centenarians were historically subject to strong selection pressures, including war, post-war famine, and widespread infectious diseases. Their relative isolation from immigration may have contributed to a more homogeneous genetic background [25]. A similar situation was observed in the Galician centenarian population, particularly in the province of Ourense.

In recent years, further research has identified five so-called “Blue Zones”—regions of the world where an unusually high proportion of individuals reach their 90s and 100s in good health; these areas are considered paradigms of healthy ageing. They include Okinawa (Japan), Ikaria (Greece), and Sardinia (Italy), among others [25]. Extensive research on centenarians in these zones has examined environmental, nutritional, sociocultural, and genetic influences on longevity. In Okinawa, a low-calorie, antioxidant-rich diet, combined with physical activity and strong social ties, has been documented. In Sardinia and Ikaria, diets based on local produce, regular physical activity, and low stress levels have similarly been noted [26].

Recent epigenetic studies—particularly in Okinawa and Sardinia—have sought to understand how environmental and lifestyle factors may modulate gene expression without altering the underlying DNA sequence [27]. These investigations have identified epigenetic signatures associated with reduced inflammation, improved cellular repair mechanisms, and more effective responses to metabolic stress. This suggests that longevity in these regions is not solely attributable to inherited genetic factors but rather to the continuous gene–environment interaction across the lifespan.

These ‘Blue Zones’ are characterised by the consumption of minimally processed foods, often sourced from self-sufficient gardens [28]. The consumption of ultra-processed food (UPF) is associated with an increased incidence of chronic diseases such as obesity, diabetes, cardiovascular disease, and certain cancers—leading causes of death worldwide. Studies involving over one million individuals confirm that high UPF intake increases the risk of heart disease and stroke [29,30].

Galicia—a region located in the north-west of Spain—follows a Southern European Atlantic Diet (SEAD) pattern and is the Spanish autonomous community with the highest consumption of fresh food and the lowest consumption of processed food [31]. This is a highly relevant finding, given that Spain is among the countries with the highest life expectancy in the world. Notable similarities can be observed between the diets of centenarians in Ourense and those in the Blue Zone of mountainous Sardinia during the mid-20th century. Both regions traditionally consumed more animal-based foods than neighbouring areas. Around 1950, Sardinia’s diet improved with an increased intake of fruits and vegetables and a moderate consumption of meat; this is similar to post-war Galicia [32], where fish and processed dairy products became more common. These dietary changes may have benefited the health of the elderly population [33].

The Mediterranean diet, as observed in this study, is enriched by the inclusion of potatoes, which provide carbohydrates and potassium with fewer calories than pasta or rice. Also noteworthy—similar to the Sardinian diet—is the intake of polyunsaturated fatty acids, primarily sourced from vegetable oils, nuts, seeds, and to a lesser extent, legumes, all of which contribute to an improved lipid profile [31].

The SEAD is increasingly recognised as an effective dietary pattern for preventing certain chronic diseases, as supported by numerous scientific publications and some clinical studies [34,35,36]. A major study published in 2021 associated the Southern European Atlantic Diet with reduced mortality from cardiovascular diseases and cancer across populations in Southern, Central, Eastern, and Western Europe [37]. These associations were of a similar magnitude to those observed for other well-established healthy dietary patterns. Regarding the consumption of SEAD food groups, the study’s findings were consistent with the dietary habits reported by centenarians in Ourense. Most participants consumed whole grain bread less than once a week; fresh fish, red meat/pork products, vegetable soup, and potatoes between one and six times per week; and dairy, legumes, and vegetables on a daily basis [37]. This adherence to SEAD principles may be one of the key factors contributing to the high number of centenarians in certain areas, such as Ourense.

Although numerous studies in the literature explore lifestyle and its relationship with centenarian longevity [38], few address the phenomenon from both quantitative and qualitative perspectives. Moreover, no such studies have yet been conducted in the region of Ourense, which, with centenarians accounting for 0.123% of its total population, holds the highest proportion of centenarians among Spanish provinces [39].

Therefore, the primary objective of this study is to analyse, for the first time, the main factors potentially associated with healthy centenarian longevity—defined as reaching this age with mild or no cognitive decline and a low or absent degree of dependency. As a secondary objective, the study aims to describe the factors that may be associated with centenarian longevity (whether healthy or not) within the province of Ourense.

## 2. Materials and Methods

### 2.1. Design, Participants and Selection Criteria

An observational, cross-sectional, and analytical qualitative and quantitative study was developed. According to data from the Galician Institute of Statistics (IGE), a total of 261 individuals aged 100 or over were identified. Based on this population, a sample size of 156 was calculated, using a 95% confidence level and a 5% margin of error. From this sample, 25 participants were randomly selected for a more detailed qualitative study involving the transcription and analysis of their narratives.

- Inclusion Criteria: Individuals aged 100 years or older having lived most of their life (more than 50%) in the province of Ourense. In cases of cognitive decline, individuals with a suitable informant available for an interview; a suitable informant is defined as a family member or caregiver (formal or informal) who knows the participant’s life history in detail.
- Exclusion Criteria: Refusal to participate in the questionnaire.

### 2.2. Variables and Instruments

The main study variables are detailed in Section S1, Table S1 (Supplementary Material), and include the following:

- Longevity calculations (for the province and each municipality): To minimise the impact of major demographic phenomena such as declining birth rates and migration, two indices have been used: (A) the Centenarian Index (CI), defined as the ratio of centenarians to individuals over 90 years old; and (B) the Longevity Index (LI), defined as the proportion of individuals over 90 years old relative to those over 65. These data were calculated based on figures from the Galician Institute of Statistics (*Instituto Galego de Estatística*, IGE).
- Socio-demographic variables: Age, sex, residence, marital status, number of children, level of education, and type of housing.
- Environmental variables: altitude: Classified as low (0–300 m), medium (300–600 m), or high (>600 m), annual average temperature and precipitation; UNESCO heritage area status: Protected or non-protected (data obtained from the National Geographic Institute and the Ministry for Ecological Transition); exposure to radon in the primary residence, defined as the place where the individual has lived the longest, and classified into Zones I, II, and III (based on information from the Ministry of Health); thermal soil presence in the area; and exposure to thermal waters in the primary residence.
- Healthy longevity variables: Cognitive assessment, applying the Pfeiffer test (validated for cognitive evaluation, Table S3) [40], and functional assessment, using the Barthel test (validated for functional evaluation, Table S4) [41].
- Longevity-related dimensions: Body Mass Index (BMI); family history; diet (questions adapted from the Food Quality Survey of the Elderly and the Survey on Dietary Habits of the Adult Galician Population, 2007); current health status (self-reported health and number of medications taken); sleep quality (questions adapted from the Pittsburgh Sleep Quality Index and the Oviedo Sleep Questionnaire); physical activity (based on the Global Physical Activity Questionnaire-GPAQ); intellectual activity; perceived stress (adapted questions from the Spanish version of the Perceived Stress Scale (PSS) [42]; occupational/employment dimension; perceived social support (questions adapted from the MOS Social Support Survey and the Duke-UNC Functional Social Support Questionnaire); Resilience (questions adapted from the Connor-Davidson Resilience Scale); personality traits (questions adapted from the NEO-PI-R test); spirituality (questions adapted from the Meaning in Life Scale; marital satisfaction (question taken from the Relationship Assessment Scale (RAS) by Hendrick [43]; life satisfaction (questions adapted from the Satisfaction with Life Scale—SWLS); self-perception of longevity (ad hoc questions to explore individuals’ attributions regarding their own longevity).

### 2.3. Procedure

- The heads of each public Primary Care service in the province of Ourense and/or their nursing coordinators were contacted. The study was presented to them, and they were invited to participate as collaborating researchers. These individuals then contacted the General Practitioners and nurses within their respective services, who, being familiar with the patients under their care, initially informed the centenarians about the project. If a centenarian expressed interest in taking part, the General Practitioner or nurse provided them with the contact details of the principal research team or, if preferred, offered to have the principal research team initiate contact. For institutionalised centenarians, the same procedure was followed through the Primary Care General Practitioner and/or nurse, or through the medical or nursing staff of the institution, if they were the designated professionals. These professionals contacted the centenarian and/or a family member and followed the same protocol.
- A member of the research team either received a phone call from the participant/family member or made the call (in cases where this was the preferred method) to explain the objective of the study, its methodology, and what participation would entail.
- For those who ultimately agreed to participate, an appointment was scheduled. The meeting location was either the health centre, a community facility, or the participant’s home. One of the interviewers, a member of the research team, provided detailed in-person information and handed the participant the study information sheet (Section S2) and the informed consent form (Section S3); if the participant agreed, the interview was conducted. Each interview lasted approximately 60 min.
- If the participant did not meet the required cognitive conditions, the procedure was discussed with their legal representative, who could either act as the informant or designate another person for that role.
- Data collection was carried out using a questionnaire (Section S4), which consisted of a semi-structured interview combining open-ended, closed, and mixed-format questions. Each item began with an open question to allow the participant to share their perspective, followed by a set of predefined response options. This questionnaire was developed ad hoc, adapting items from validated instruments used to explore the various dimensions of interest (Section S1), and based on tools found in the relevant literature, such as the *Chinese Longitudinal Healthy Longevity Survey Biomarkers Cohort* (HLSBC) [44] and the Newcastle 85+ Study [45], adapted to our cultural context.

The interviews were audio-recorded for subsequent qualitative analysis. If the centenarian had any physical or cognitive limitation that made direct interviewing difficult, the interview was conducted with an informant (a family member or caregiver with detailed knowledge of the participant). Interviews were conducted either in the participant’s home or, if preferred, at an alternative location such as a health centre or a community facility.

The interviewers, a maximum of four, were members of the research team; they received prior instruction and training, and endeavoured to document, as far as possible, the participant’s gestures and non-verbal communication for qualitative analysis. All interviewers adhered to ethical standards and signed a confidentiality agreement.

### 2.4. Data Analysis

The analyses were performed using the Statistical Package for Social Sciences (SPSS), version 29.0.

- Quantitative Analysis: Quantitative variables were expressed as a mean and standard deviation when they followed a normal distribution, or as a median and interquartile range if they were non-Gaussian. Normality tests included the Kolmogorov–Smirnov and Shapiro–Wilk tests. A descriptive statistical analysis of the variables was carried out. Multivariate analysis and logistic regression were also conducted to identify which variables acted as independent factors associated with healthy longevity.

The analyses were conducted using the Statistical Package for the Social Sciences (SPSS), version 29.0. Quantitative variables are expressed as the mean and standard deviation, provided they follow a normal distribution. A descriptive statistical analysis of the aforementioned variables was carried out. In addition, a bivariate analysis was performed between the dependent variables (cognitive impairment and level of dependency) and all other variables, using the Chi-squared test to assess statistical significance, and Fisher’s exact test when expected frequencies were low (<5). The odds ratio (O.R.) was also calculated to estimate the strength of the association between variables.

- Qualitative Analysis: A thematic qualitative analysis was carried out using a mixed categorisation approach (both pre-established and inductive), with the aim of identifying emerging factors not previously reported in the available literature, which might arise from the accounts of centenarians or their informants.

The data analysis began with the verbatim transcription of the interviews, which had been previously recorded. Following transcription, a critical review of the collected data was conducted. The transcripts contained no personally identifiable information. The data were coded, and only members of the research team had access to the coding system.

An initial phase of organisation, processing and analysis was performed using the software Atlas.ti. This was followed by a stage focused on identifying and interpreting the content. This involved reading, reflecting, writing, and rewriting. Subsequently, data analysis was performed by developing “categories” that matched the information received. The interpretation was carried out independently by two researchers. Table S2 (Supplementary Information) presents the coded discourse according to the main thematic categories.

### 2.5. Ethical Considerations

This study was approved by the Research Ethics Committee of Pontevedra-Vigo-Ourense, part of the Galician Health Service (SERGAS), under Registration Code no. 2023/399. The study was conducted in accordance with Good Clinical Practice guidelines, the fundamental ethical principles established in the Declaration of Helsinki and the Oviedo Convention, and ensures the protection of personal data processing, as stipulated in the Spanish Organic Law 3/2018 of 5 December, on the Protection of Personal Data and Guarantee of Digital Rights, as well as the applicable Spanish legislation governing research. The study commenced following a favourable opinion from the Ethics Committee and authorisation from the Management of the Healthcare Area of the city of Ourense, Verín, and O Barco de Valdeorras.

With regard to data processing, audio recordings were transcribed into text. These transcriptions did not include any personally identifiable information, which was pseudonymised and coded. Care was taken during the writing and dissemination of results to avoid including any personal data that could lead to participant identification. All transcripts and recordings were destroyed at the end of the study. A copy of the recording was offered to each interviewee.

## 3. Results and Discussion

### 3.1. Location of the Study Sample

**Figure 1:**
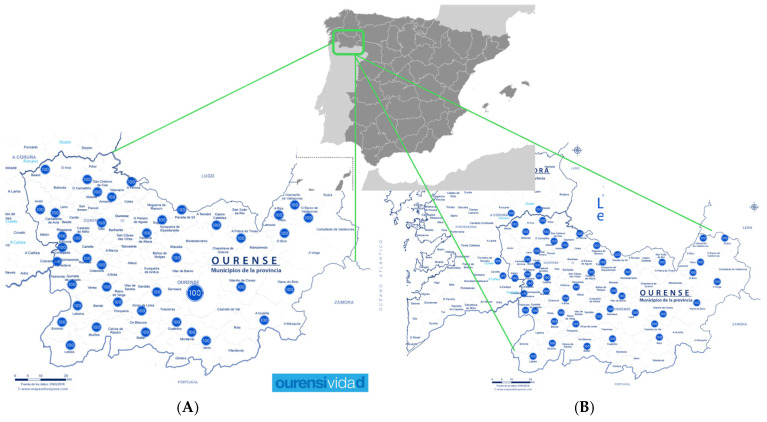
(**A**) Distribution of the interviews conducted with centenarians in Ourense, (**B**) Areas of exceptional longevity in the province of Ourense.

The results of the interviews conducted in the longevity hotspots of the province of Ourense can be seen in Figure 1.

As shown in Figure 1A, the distribution of the interviews is homogeneous throughout the territory of the province of Ourense. Interviews with centenarians from the western, central, and eastern areas were carried out in this study. It is important to note that the western area shows a higher density of centenarians. Figure 1B displays the areas of exceptional longevity. This map more clearly highlights the higher concentration of centenarians in the western part of the province.

### 3.2. Sociodemographic, Clinical, and Functional Characteristics of the Sample

**Sex of the participants.** The sex distribution of the participants shows a majority of women (73%), with 84 women and 31 men, representing 27% of the sample. This bias toward a higher female representation is related to the greater life expectancy of women compared to men, which is consistent with observations in many populations. However, the male representation in our sample is higher than in the centenarian population of Galicia, which may be because a greater number of women declined to participate in the study.

**Figure 2:**
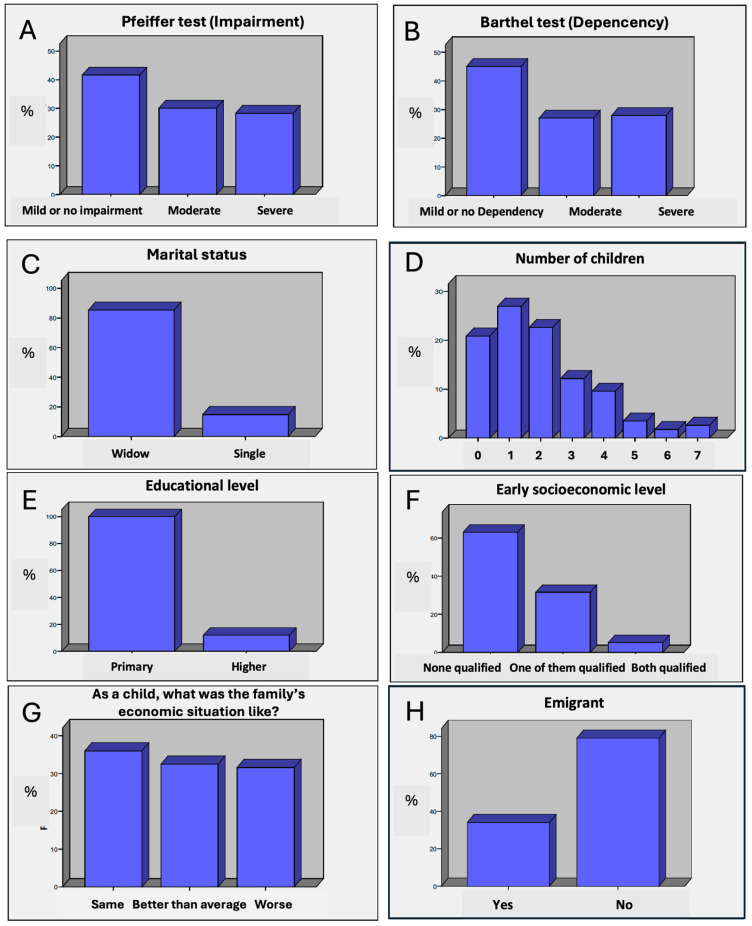
Evaluation of the (**A**) cognitive status (degree of impairment) according to the Pfeiffer Test, (**B**) degree of dependency according to the Barthel Test, (**C**) marital status, (**D**) number of children, (**E**) educational level, (**F**) early socioeconomic level, (**G**) family economy, and (**H**) emigration.

**Cognitive function according to the Pfeiffer Test.** The Pfeiffer test (Section S5, Table S3) was used to assess the degree of cognitive impairment among participants (Figure 2A). The results showed that 41.6% of respondents had no dementia or only mild cognitive impairment, 30.1% had moderate impairment, and 28.3% were affected by dementia. These results are not representative of the prevalence of cognitive impairment in the general population, as there was a greater tendency for participation among individuals without cognitive impairment. Nevertheless, from a qualitative perspective, it can be reported that participants with mild cognitive impairment (and even some with severe impairment) were able to maintain a coherent discussion when asked about their past. The relationship between the degree of cognitive impairment and other variables, such as marital status or number of children, may provide valuable insights into the factors influencing individuals’ cognitive health.

**Degree of Dependency according to the Barthel Test.** The Barthel Test (Section S5, Table S4) was used to assess the participants’ capacity to perform activities of daily living. The results indicate that 37.8% of participants had mild or no dependency, 32.7% had moderate dependency, and 29.6% had severe dependency (Figure 2B). Among dependent individuals, a noticeably lower level of satisfaction was evident in their discourse, with more frequent expressions of complaint. For these individuals, extreme longevity is often not perceived as an advantage.

**Marital status.** A total of 85.2% of the respondents were widowed, while 14.8% were single. The high proportion of widowed individuals reflects the survival of one partner into old age, which may be associated with various effects on emotional wellbeing and quality of life. This finding underscores the importance of social support, as widowhood can increase vulnerability to psychological conditions such as depression. Marital status also influences health-related decision-making, as being widowed or living alone can pose greater challenges in terms of access to medical care and familial support (Figure 2C).

**Number of children.** Regarding the number of children, most participants reported having between 1 and 2 children; 27.0% had one child, and 22.6% had two children (Figure 2D). The result is surprising, given that the literature suggests that the number of children can influence family support networks, such that people with more children tend to have a broader support network, which can be beneficial for care and attention in old age. However, the fertility rate of centenarians stands at a median average of 2 children, which is below the generational replacement rate.

**Educational level.** The educational level of the participants is another relevant factor in the analysis. The vast majority of respondents (89.3%) had completed only primary education, with just 10.7% pursuing studies beyond this level (Figure 2E). This low educational level could have implications for individuals’ ability to access health information, make informed decisions, and maintain healthy behaviours. Education is closely related to people’s ability to manage their health and make choices that support healthy aging.

**Early socioeconomic level.** The socioeconomic level during childhood, as we have seen in the Introduction, is a determining factor for longevity. Assessing this dimension is complex. The narratives of the centenarians allowed us to understand that those whose parents, or at least one of them, had a skilled profession or trade enjoyed greater abundance. It is true that most of the parents who lived in rural areas were engaged in farming, but some of them combined agricultural work with certain specialised professions such as carpenter, blacksmith, seamstress, etc. In these cases, in addition to greater financial ease, there was a broader awareness of the surrounding reality, as they travelled to other towns, or even provinces, to carry out their work; also, they had a greater interest in their children learning professions that would enable them to sustain a welfare economy, not just subsistence. And, above all, there was a way to convert work into money, which allowed access to market goods and products or enabled them to face complex health problems. Nevertheless, as shown in the following table, cases in which none of the parents performed skilled work predominate (72%) (Figure 2F).

Another aspect used to assess early socioeconomic level is the family economy. In this case, evaluating it through the centenarians’ narratives must be achieved by using their own environment as a comparative model. Here, the distribution is balanced across the three categories (Figure 2G).

**Emigration.** The percentage of centenarians who emigrated to other countries stands at 30%, which is close to the emigration figures for the general population of Galicia, which throughout the 20th century ranged between 20% and 40% (Figure 2H).

**Centenarians’ condition based on polypharmacy.** This is defined as the daily intake of five or more different medications. Based on this criterion, centenarians were classified according to the age at which they began taking five or more medications.

**Figure 3:**
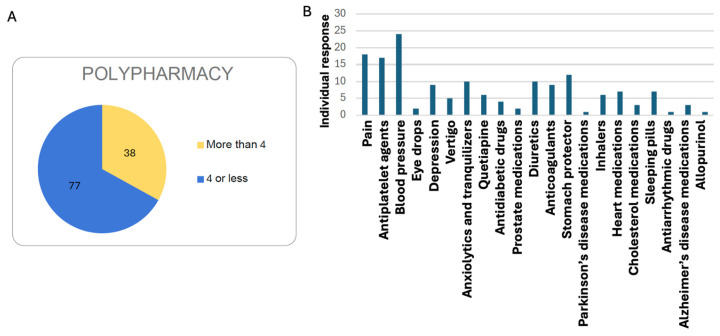
(**A**) Centenarians experiencing polypharmacy (number of drugs taken per centenarian); (**B**) classification of the medications taken by the centenarians of Ourense.

The data collected from the surveys of centenarians in Ourense is striking in this regard. If we define a centenarian experiencing polypharmacy as someone who takes more than four medications daily, we observe that the number of non-polypharmacy individuals is twice that of those affected by polypharmacy. This suggests that a key characteristic among centenarians in Ourense is the delayed onset of chronic diseases. Figure 3A illustrates the distribution of centenarians undergoing polypharmacy.

**Medication.** A total of 23 centenarians either take no medication or only one. Since most of the centenarians are women, medications more commonly associated with men appear less frequently. The most commonly used (or previously used) medications (Figure 3B) are those related to blood pressure and antiplatelet agents—in other words, all associated with the cardiovascular system. These are followed by medications used to treat pain, with paracetamol being the most frequently used. Most have been taking their medication for 10 to 15 years, and they commonly report having started treatment around the age of 85.

This aligns with the definition of “delayer” centenarians in terms of the onset of medication use. For this reason, with regard to chronic conditions such as cardiovascular disease, diabetes, hypertension, and hypercholesterolaemia, most participants exhibit a very low incidence, which is an illuminating finding.

Only a minority (12 individuals) of the 115 interviewed reported having diabetes, with most cases diagnosed in advanced age (Figure 3A). It is possible they lived through a time when diabetes was not afforded the same level of attention as it receives today. As for hypertension, around one-third of respondents (36 out of 115) reported being hypertensive. Regarding hypercholesterolaemia, 15 participants reported having it, while 84 stated that their cholesterol levels were normal. A small number did not respond to this question.

**Figure 4:**
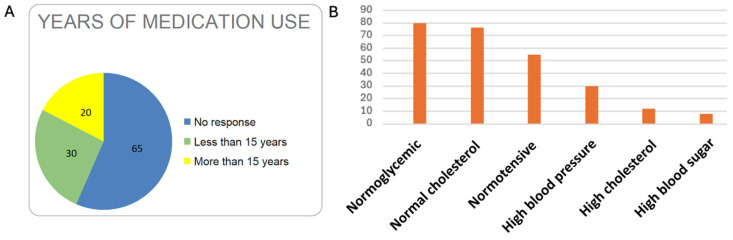
(**A**) Length of time on medication and (**B**) chronic diseases and frequency (%) among the centenarians in the province of Ourense.

**Duration of medication use.** Only 20% of centenarians received medication for more than 15 years, while another 30% received medication for less than 15 years. In this section, 65% either did not respond to the question or could not recall it accurately (Figure 4A).

**Chronic diseases.** One of the most interesting findings of the study is the low prevalence of chronic diseases among the centenarians in the province of Ourense (Figure 4B). Indeed, 80% were normoglycaemic and 75% had no cholesterol-related issues. This is closely linked to high longevity, as these variables are inversely proportional.

**Figure 5:**
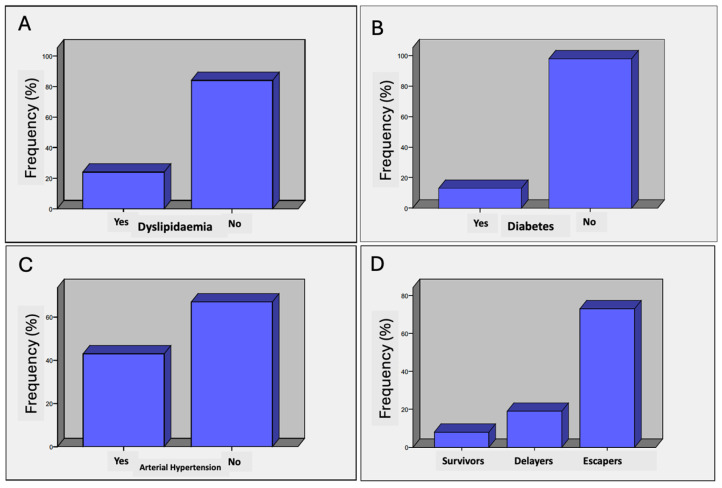
(**A**) Presence of dyslipidaemia; (**B**) percentage of diabetics; (**C**) centenarians with arterial hypertension; (**D**) percentage of escapers, delayers, and survivors among the centenarians.

**Dyslipidaemia.** In total, 20.9% of the centenarians reported problems with dyslipidaemia (Figure 5A). This is a slightly lower prevalence than that of the general population in Galicia (25%). Therefore, dyslipidaemia does not appear to be a distinguishing factor between less and more long-lived populations.

**Diabetes.** In total, 11.7% of the centenarians reported having diabetes, all of whom had type 2 diabetes (Figure 5B). The prevalence of this condition in the general population is around 7%, but among people over the age of 65, it reaches 26%. Diabetes does appear to be a differentiating factor. The lifestyles of centenarians—particularly their carbohydrate consumption patterns, tendency towards normal body weight, and levels of physical activity—may have acted as protective factors against insulin resistance and diabetes.

**High blood pressure (hypertension).** In total, 32.7% of participants reported having experienced high blood pressure, while 60.9% stated they had never suffered from it (Figure 5C). Hypertension is a common condition in old age and is associated with a higher risk of cardiovascular disease, stroke, and other serious health problems. The prevalence of hypertension in the general adult population is around 30%, but in those over the age of 65, it is at least 50%. Hypertension likely acts as a differentiating factor, and its absence may be associated with greater longevity, according to our findings.

Most of our centenarians belong to the “escaper” category, accounting for 73% of valid cases (Figure 5D). It should be noted that several participants did not remember the age at which they started to be daily medicated, so they were marked as missing by the system. Even if we assigned that 13% to either survivors or delayers, the dominant percentage of “escapers” would still be 63%. Among respondents (*n* = 56) who answered the question about the type of medications they take (a response often provided by their caregivers), it is evident that the most used drugs are those related to blood pressure and antiplatelet agents, all of which are associated with the cardiovascular system.

### 3.3. Nutrition: Dietary Analysis

#### 3.3.1. Amount of Food Consumed per Day and Number of Meals per Day

**Figure 6:**
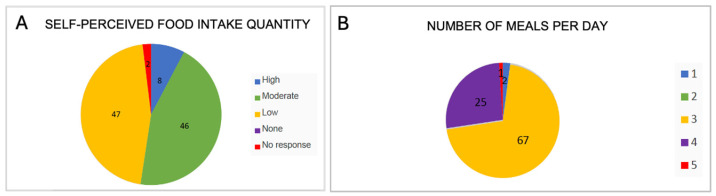
(**A**) Assessment of the amount of food consumed and (**B**) number of meals per day. The numbers indicate individual responses.

In response to the question “Did you eat a lot or a little?”, the answers were relatively balanced between those who reported consuming a large or moderate amount of food and those who stated they ate little (Figure 6A). This suggests a general moderation in food intake, which aligns with their physical constitution, as the majority were of normal weight.

Most centenarians from Ourense usually had three meals a day (Figure 6B). They reported eating between three and four times a day, with only a few stating they ate just once or twice daily. Half of the centenarians served themselves small portions, while the other half ate fairly large amounts. A general tendency toward smaller food portions was observed. Over 70% of participants had three meals a day, followed by 26% who reported four meals daily. It was also common to have between three and four meals, often including an afternoon snack. Eating just one or five times a day was the least frequent pattern mentioned in the interviews.

The four-meal pattern typically included an afternoon snack or a mid-morning snack when they got up early to work in the fields or tend livestock; these are two meals that were very typical in the past. The most common breakfast reported was milk with bread (sometimes adding coffee, cocoa, or chocolate), or broths and soups. Some participants (5 cases) also mentioned corn cake or cornmeal, three mentioned coffee liqueur, one had aguardiente (a strong spirit) with bread, another mentioned wine-soaked bread, and one included oats, fruit, and some nuts.

#### 3.3.2. Consumption of Greens, Vegetables, Legumes Fruits

**Figure 7:**
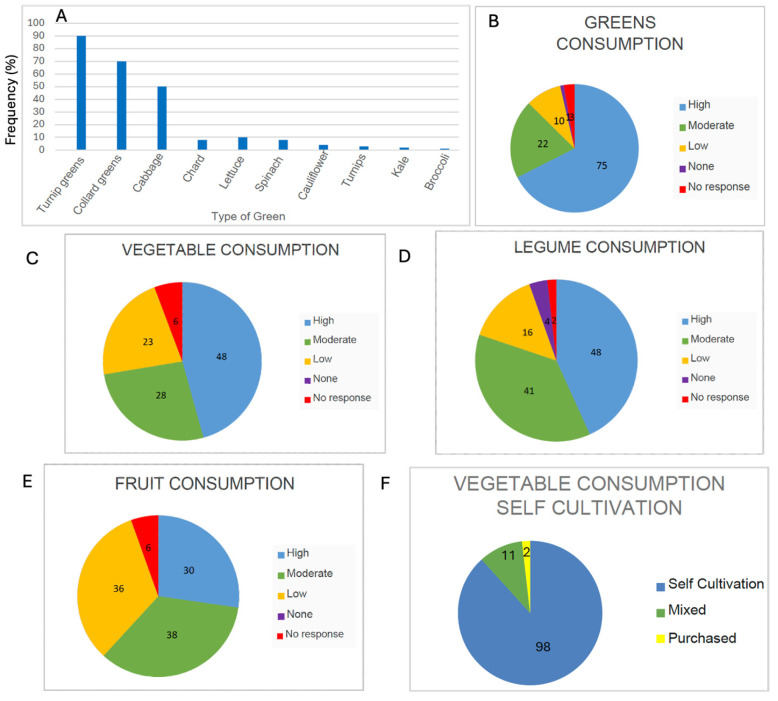
(**A**) Types of vegetables, (**B**) level of vegetable consumption, (**C**) vegetable intake, (**D**) legume consumption, (**E**) fruit consumption, and (**F**) consumption of self-grown garden produce. The numbers indicate individual responses.

The centenarians of Ourense stand out for being heavy consumers of leafy greens, either because they grow them in their own gardens or receive them from neighbours in rural areas. They consume many types of greens depending on the season, being particularly fond of collard greens, turnip tops, broccoli rabe, and cabbage—all vegetables from the *Brassica* genus (Figure 7A,B). Ourense is one of the Spanish provinces with the highest consumption of this type of green vegetable. Brassica vegetables are known for their anti-cancer, anti-inflammatory, and antioxidant properties, thanks to their bioactive compounds such as sulforaphanes [31,46]. These foods are mostly self-grown, as home cultivation is a recurring pattern among nearly all interviewees. Indeed, Ourense is the Spanish province with the highest consumption of home-grown produce [47].

Regarding other vegetables, 72% of participants reported a high level of consumption (either “a lot” or “quite a bit”), particularly when in season (Figure 7C). Tomatoes and onions were the most frequently consumed varieties.

With respect to legumes, 51 centenarians reported consuming a large amount, while 34 indicated moderate consumption; eleven reported low intake, and only two stated that they did not consume legumes at all. Thus, the vast majority (85 out of the 98 centenarians interviewed) reported a high intake of legumes (Figure 7D). The most commonly consumed varieties were lentils and broad beans.

As for fruit, the quantity was more notable than the variety. Most participants reported eating a couple of pieces of fruit regularly (68 of 110) (Figure 7E), typically distributed among apples, pears, grapes, cherries, quince, or plums. It is worth noting that some interviewees mentioned eating kiwi, and this highlights a current trend, as kiwi has become well-established in the region. Galicia is now one of the main kiwi-producing regions in Spain.

Concerning self-grown produce, the data are striking since the vast majority (98 of 111) of centenarians have a vegetable garden for personal consumption, where they grow their own vegetables and greens (Figure 7F). Ourense is also the Spanish province with the highest number of self-sufficient vegetable gardens per capita [48]. As previously mentioned, some of the most commonly grown vegetables belong to the Brassica genus, such as turnip greens, young turnip tops, cabbage, and the traditional cabbage.

#### 3.3.3. Meat and Fish Consumption

**Figure 8:**
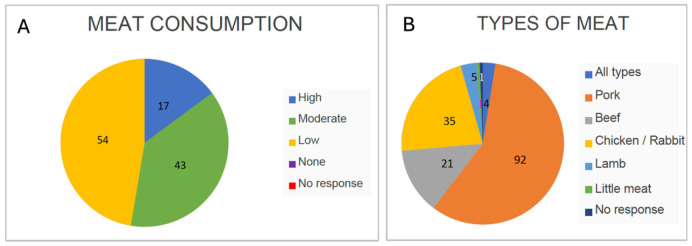
(**A**) Level of meat consumption and (**B**) types of meat consumed by the centenarians of Ourense. The numbers indicate individual responses.

In general, 53% of respondents reported eating a lot or quite a bit of meat, typically with a frequency of 2–3 times per week. Additionally, 47% stated they ate little meat, though very likely on a daily basis in small amounts (Figure 8A). When referring to meat, most participants did not include cured meats in this category. However, it is important to consider them, as traditional—and annual—pig slaughter often led to the high consumption of homemade cured products such as *chorizo* and *salchichón*.

As previously mentioned, among the types of meat consumed, pork was the most frequent (58%), followed by chicken/rabbit (22%), beef (13%), and finally lamb (3%) (Figure 8B). The consumption patterns likely reflect the availability of each type of meat and the domestication of animals.

**Figure 9:**
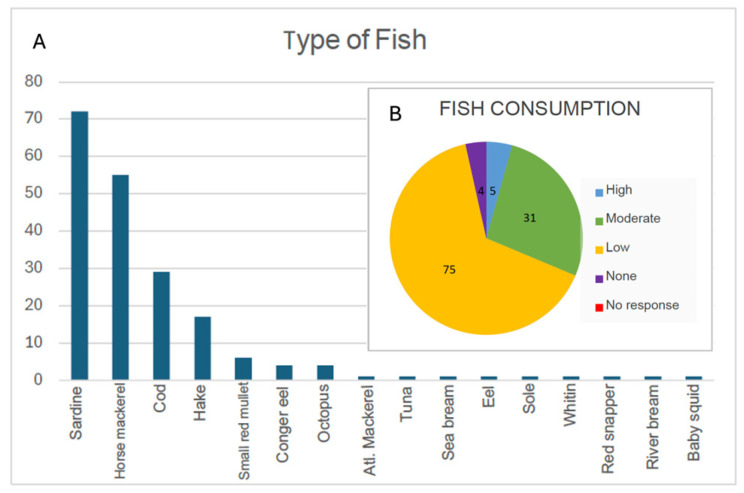
(**A**) Types of fish and (**B**) level of fish consumption among the centenarians of Ourense.

Fish was generally less consumed than meat; those who did eat it did so 1 to 2 times per week. Notably, the centenarians primarily consumed oily fish, with sardines and horse mackerel being the most common, due to their affordability and accessibility. Oily fish is a key element of the Atlantic Diet and is rich in omega-3 fatty acids, making it highly recommended for the prevention and treatment of cardiovascular diseases—one of the leading causes of death worldwide—thus contributing to longevity. Among oily fish, trout was also mentioned. Other non-oily fish that were consumed included hake and cod. Additionally, a few centenarians (4%) reported eating octopus (Figure 9A). The rather moderate or low consumption of fish (Figure 9B) is somewhat surprising, as it deviates from the high fish consumption pattern typically observed in the SEAD. However, this is understandable given that the whole province is inland, with no access to the sea, and less access to fish.

#### 3.3.4. Consumption of Sweets, Bread, and Potatoes

**Figure 10:**
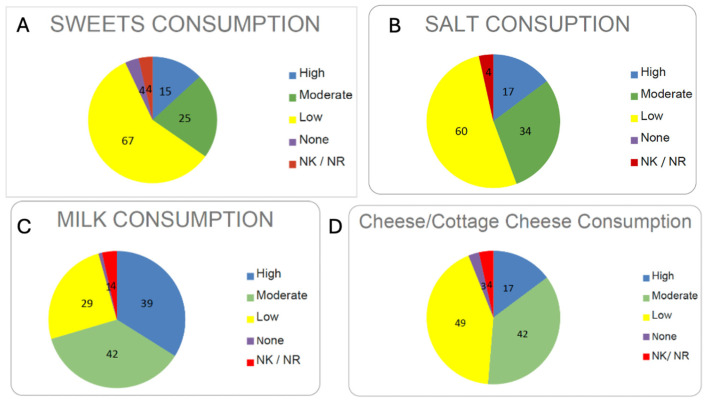
Evaluation of the (**A**) sweets consumption, (**B**) salt, (**C**) milk, and (**D**) cheese/cottage cheese. [NK: No Knowledge; NR: No Response]. The numbers indicate individual responses.

The consumption of sweets among the centenarians of Ourense is low (Figure 10A). The interview focused on simple carbohydrates such as honey, sugar, or desserts. There was a strong consensus toward “low consumption,” as shown in Figure 10.

By food groups, a daily intake of carbohydrates was observed, with bread and potatoes being predominant. Overall, there is a high consumption of bread and potatoes, with 53% of respondents reporting eating “a lot” and 31% reporting “quite a bit”. In some cases (three participants), bread consumption was reported to be higher than that of potatoes.

#### 3.3.5. Consumption of Salt, Milk, Cheese, and Cottage Cheese

Average salt intake is low (Figure 10B), which aligns with one of the main pathologies found in the medication data: drugs prescribed for cardiovascular diseases.

On the other hand, milk consumption is significant (Figure 10C). Of those surveyed, 81 centenarians reported a good level of milk consumption, while only 30 said they consumed little or none. In many cases, the milk was homemade, sourced from cows they owned or from a neighbour’s farm.

The consumption of cheese or cottage cheese is lower compared to milk (Figure 10D). A total of 59 participants reported consuming a lot or quite a bit of cheese/cottage cheese, whereas 52 said they consumed little or none. This may be due to the greater effort required to produce these foods or their potential cost, leading to a preference for milk over its derivatives.

#### 3.3.6. Consumption of Wine and Distilled Beverages

**Figure 11:**
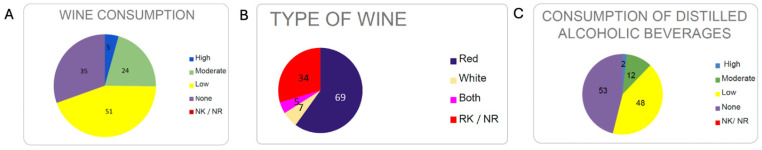
(**A**) Wine consumption, (**B**) types of wine, and (**C**) consumption of distilled beverages among the centenarians of Ourense. [NK: No Knowledge; NR: No Response]. The numbers indicate individual responses.

Alcohol was not, and is not, predominantly consumed among our centenarian interviewees. An overwhelming 75% of respondents reported little to no alcohol consumption (Figure 11A). Among the rest, only one centenarian reported drinking distilled spirits other than traditional liqueurs with Protected Designation of Origin (such as coffee or herbal liqueurs). Fourteen centenarians reported drinking some coffee or herbal liqueur, and the remaining ones mentioned having a glass of red wine with meals. Overall, 30% of the interviewees reported not drinking any alcohol at all.

**Type of wine.** The wine is usually homemade, and in most cases, red wine is preferred (Figure 11B). It is worth noting that among the centenarians of Ourense, wine consumption was low and typically associated with a celebration or a small glass at lunch.

**Consumption of distilled beverages**. When it comes to distilled beverages, interviewees were very clear in their position: 101 out of 115 reported not drinking or drinking very little (Figure 11C). In most cases, the only exception was homemade coffee or herbal liqueur.

#### 3.3.7. Importance of Drinking Water

**Figure 12:**
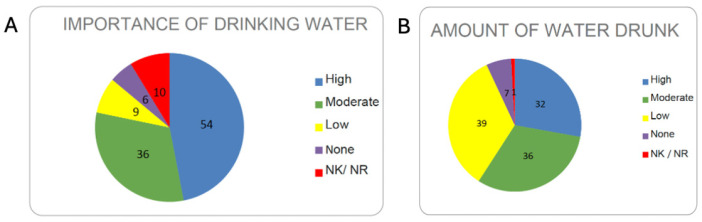
(**A**) Importance attributed to water consumption by centenarians; (**B**) self-reported level of water intake among centenarians [NK: No Knowledge; NR: No Response]. The numbers indicate individual responses.

**Water consumption.** Centenarians in Ourense place high importance on water consumption, with a strong majority giving it a positive value. Of the 115 surveyed, 90 affirmed that drinking water is very important, while 15 stated otherwise; the rest referred to what others might say (Figure 12A).

**Amount of water consumed.** In terms of quantity, high and moderate water consumption was more common than low intake. The responses were consistent with the previous question about the importance placed on water consumption—they reported drinking a lot or quite a bit of water (Figure 12B).

**Figure 13:**
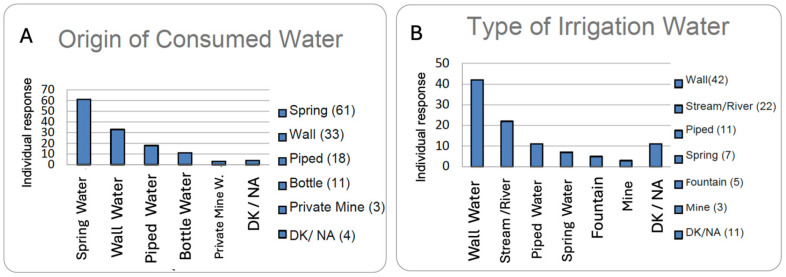
(**A**) Sources of drinking water and (**B**) type of irrigation water [NK: No Knowledge; NA: No Answer]. The numbers indicate individual responses.

**Source of drinking water.** This section reflects the quality of water in Ourense, as most respondents (90%) reported drinking water from springs, fountains, wells, or local piped supply, as shown in the graph. This supports the idea of high-quality water emerging from underground aquifers. The rest (~10%) reported consuming water from mines or bottled water (Figure 13A).

**Type of irrigation water.** It is important to know the origin of the water used to irrigate the self-sufficient home gardens, which are so characteristic of the province of Ourense. The irrigation water mainly comes from wells and small streams; it also comes from piped sources, springs, and fountains, ensuring very high irrigation water quality (Figure 13B).

Regarding the quality of the water consumed by centenarians, whether for drinking, irrigation, or cooking, it mainly came from natural sources such as springs or wells. These waters were typically soft and low in mineral content, yet rich in key minerals like lithium, calcium, and magnesium, which are closely linked to bone health and mood regulation. However, traces of nitrates were also detected due to agricultural and livestock activities. From a chemical perspective, most of the water consumed by centenarians was of good quality.

The physicochemical properties and composition of these waters are strongly influenced by the predominant lithology in Galicia, which consists mainly of crystalline silicate rocks. The waters are typically low-mineral, sodium bicarbonate types, with a significant presence of silica, a mineral associated with benefits for bone and connective tissue health. All samples contained silica, with an average concentration of 10 mg/L, a noteworthy level due to its potential positive effects on musculoskeletal health. These are soft waters, with low to very low mineralisation (a median of 63 mg/L of dissolved solids), and are predominantly bicarbonate in composition. Sodium is present at an average concentration of 7.3 mg/L, and the waters also contain small amounts of calcium and magnesium. All samples showed silicon concentrations around 10 mg/L, along with trace amounts of lithium and fluoride. The aforementioned sodium levels allow these waters to be recommended for low-sodium diets (Na < 20 mg/L), such as the DASH diet, which is commonly used to manage hypertension, a major public health issue, particularly in Western societies [49]. Table 1 provides a comparison of the most prominent and distinctive nutritional aspects among the dietary patterns of Okinawa, Sardinia, and Ourense.

The culinary culture of Okinawa has three main characteristics: (i) a focus on a combination of foods rather than isolated ingredients, with particular attention to their synergies; (ii) mixed dishes that include both plant- and animal-based products (e.g., garlic, carrots, and pig liver, or vegetables and freshwater fish); and (iii) a tendency to prioritise locally available foods over those prescribed in classical Chinese medical texts. Particularly noteworthy is the intelligent use of all edible parts of the pig—including the tail, feet, ears, internal organs, blood, and fat—a tradition that coincides with that of Ourense, where the most typical custom, known as la matanza del cerdo (the pig slaughter), involves using every part of the animal [50].

The traditional consumption of various seaweed species by the people of Okinawa is ultimately attributable to historical and cultural influences. Kombu (Saccharina latissima) is one such seaweed. In contrast, in Ourense, seaweed consumption is virtually non-existent, and historically, goitre was prevalent.

Okinawans regularly consume tofu derived from soybeans. Unlike Sardinia and Ourense, the exceptional longevity of the Okinawan population has been linked to a calorie-restricted diet [51]. Among the centenarians of Ourense, tofu consumption was practically non-existent, meaning that their isoflavone intake likely came from supplementation. In animal studies, a 30–60% reduction in energy intake without malnutrition has been shown to extend lifespan. In Okinawa, diets were hypocaloric. In contrast, the diets of centenarians in Ourense were normocaloric. In Galicia, food portions tend to be generous, and there is no tradition of caloric restriction.

Cured meats are seldom consumed in Okinawa. In Ourense, the typical cured meat—chorizo—originates from the pig slaughter tradition. Meanwhile, the native population of Sardinia, a semi-nomadic community of herders, was historically forced to retreat to the central mountainous area (Ogliastra) to maintain a degree of independence, and developed a markedly different diet. This diet included a relatively high intake of animal products such as cheese, meat, and pork fat. Sourdough wheat bread was the staple food [51].

This shows some similarity to the findings from the longevity study of centenarians in Ourense, where there are five Protected Designations of Origin (PDO) for cheese, and pork is the primary source of meat in the diet. Bread, made with sourdough and yeast, also features as the main carbohydrate accompanying meals. Potato consumption is another major commonality. Ourense is known for its PDO product, Patata de A Limia. In Sardinia—particularly the longevity region of Ogliastra—this tuber is also heavily featured in local minestrone soups.

Sardinia’s typical cured meat, known as *guanciale*, is flavoured with aromatic herbs. The local minestrone (vegetable soup) commonly includes onion, fennel, carrots, and legumes (beans, broad beans, peas), along with plenty of potatoes. However, traditional minestrone was often enriched with small pieces of salted bacon. A diet high in carbohydrates has been associated with greater life satisfaction among residents of Sardinia’s Blue Zone [52].

Unlike most Mediterranean regions, the diet in Sardinia’s Blue Zone was influenced by two key factors: (i) the legacy of a pastoral culture based on animal husbandry that characterised the local population before Roman rule; and (ii) the effect of the nutritional transition (NT), which began in the mid-1950s and diversified the food supply in the latter half of the 20th century. Specifically, before the NT, the balance of plant and animal food consumption was clearly skewed in favour of the latter, especially dairy, which was the main source of protein. This may have helped preserve muscle mass among the elderly, in line with findings from the NHANES III study [52].

Similarly, high dairy intake is observed among centenarians in Ourense. However, in Ourense, it primarily comes from cow’s milk, while in Sardinia it is derived from goats and sheep. One key dietary difference between Sardinian and Ourense centenarians lies in the vegetables consumed. In Ourense, preference is given to vegetables from the Brassicagenus, such as *grelos* and *nabizas* (turnip greens), which, as shown in the graphs, are widely consumed. These vegetables have anti-inflammatory, antioxidant, and anticancer properties.

Another significant difference between the dietary patterns of Ogliastra and centenarians in Ourense concerns alcohol consumption. In Sardinia, alcohol intake follows the Mediterranean pattern—moderate consumption during meals [53]. Among centenarians in Ourense, however, alcohol intake is virtually non-existent and anecdotal at best.

### 3.4. Assessment of Longevity Determinants Beyond Nutritional Aspects [47]

The parameters explored below, which are associated with longevity but not with the nutrition of the centenarians, were also investigated and evaluated. The results are shown in Figures S1–S3 (Supplementary Material).

**Thermal water**. We observed that 83.7% of the centenarians either did not use thermal waters or used them very little (Figure S1A). This is a surprising finding in a province like Ourense, known for its thermal springs. This data can be explained through qualitative information. In their narratives, the centenarians clarify the reasons for this underuse of the resource. Many of them never went to spas because “*they were expensive and there was no money to pay for them*.”

**Physical activity.** The centenarians interviewed generally maintained active lifestyles from a locomotor point of view, with little daily sedentary behaviour (Figure S1B). A total of 94% reported being people who move around quite a lot or a great deal. Regarding physical activity, the majority of centenarians reported engaging in either very or fairly intense physical activity, with a cumulative percentage of 81.4% (Figure S1C). Only 13% reported having low levels of physical activity. Most of them carried out physically demanding work related to farming.

**Daily physical activity.** However, this physical activity was integrated into their daily routines. They generally did not engage in additional physical efforts in the form of exercise or sports. A total of 90.3% did *not* do “any” sport (Figure S1D). We can say that the centenarians developed a significant level of daily physical activity, integrated into their regular tasks, but not through planned physical exercise. A high proportion of individuals were of normal or underweight build throughout their lives (Figure S1E).

**Tobacco use.** Smoking is a well-known risk factor for many chronic diseases, including cardiovascular diseases. In the sample studied, only a small percentage of participants (8%) had been smokers, while the vast majority (92%) had not (Figure S1F). Strictly speaking, all smokers were former smokers, as none of them were current smokers, making the prevalence of active smoking in our sample effectively zero. In the general Galician population over the age of 16, the smoking prevalence ranges between 15% and 20% [48]. The proportion of former smokers in the elderly population is about 22.1% [48]. In our study, there was only one female former smoker compared to seven male former smokers. In other words, smoking has a higher prevalence in the general population than in the centenarian population. Even the rate of former smokers is lower among the extremely long-lived. Tobacco clearly appears to be an anti-longevity factor.

**Sleep patterns.** The sleep quality of the majority was very good or fairly good in up to 90.4% of the cases (Figure S1G). However, centenarians do not generally consider themselves to be “*heavy sleepers*”. Up to 60% reported feeling “*less sleepy*” than those around them. With regard to hours of sleep, it is difficult to accurately estimate how much they slept over the course of their lives, as this varied across different life stages. However, the majority (74.3%) reported having slept more than seven hours per night (Figure S1H). Furthermore, as can be inferred from the qualitative analysis of the interviews, participants tended to base their estimates on the most recent stage of life—typically from retirement onwards—when sleep routines became more flexible and less constrained by the need to wake early for work. Time spent in bed was often equated with time asleep, a factor which tends to be higher among centenarians. As such, the quantitative data are likely to overestimate the actual number of hours slept.

Only 8.8% of centenarians reported significant problems with insomnia, and when present, such issues typically appeared later in life. In total, 33.3% stated they had experienced insomnia at some point in their lives (Figure S1I). These figures are in line with the general population, where insomnia prevalence ranges from 10% to 30%. However, in the elderly population, the prevalence is between 30% and 50% [48].

**Hypnotics.** The use of sleep medication (hypnotics) at some point in their lives (including the present) is 21.2%, which aligns with the typical use among people over 65, estimated at 20–30% [48] (Figure S1J).

**Napping.** Regarding napping habits, a majority—71%—reported sleeping little or not at all during the day (Figure S1K). Napping was not a deeply rooted habit, occurring only occasionally and typically in response to fatigue or sleep deprivation. Some practised seasonal napping, particularly in summer, with fewer doing so in winter; only 28% reported napping with any regularity. In Spain and other Mediterranean countries, around 40% of the population is estimated to nap regularly [48].

The influence of sleep as a factor in longevity remains controversial. It appears that sleep patterns are likely a weak differentiating factor in extreme longevity. Although centenarians differ from the general population in terms of hours slept—an estimate that lacks precision and contradicts the fact that most report less desire or tendency to sleep than those around them—the most notable difference lies in the self-perceived quality of sleep.

The prevalence of insomnia among centenarians is similar to that of the general population, and while it may be slightly lower than in the wider elderly population, their use of sleep medication is comparable. The most striking difference is the lower frequency of daytime napping among centenarians, which challenges the perceived benefits of napping and aligns with what is suggested in the literature.

**Affability.** Based on the statements of the centenarians we interviewed, we broke down the dimension of affability into the concepts considered most meaningful. Thus, we explored affability, cordiality, and harmony. As a related and complementary concept, we also studied sensitivity, using their own expressions such as having a “*hard*” or “*soft*” heart. Focusing on friendliness, understood as the tendency to get along well with others, we observed high percentages: those who reported getting along “*very well*” or “*quite well*” with their social contacts represent a combined total of 98.2% (Figure S1L). From a qualitative perspective, affability is a very notable trait. Getting along well with others becomes almost a motto or life philosophy. M21 insists that “*The point is to get along with everyone.*” F23 says that the most important thing is “*To get along well… The family, to get along with everyone*” and for F25, what one must achieve in life is “*To get along well, that’s the main thing*”. In fact, most of the interviewees show an affable and cordial attitude toward the interviewer. They experience empathy for the suffering or death of family and friends, and some become emotional and cry when remembering them. It is true that affability has its limits, one of which is related to property. M3 says “*I had some arguments with the neighbours because they were moving my boundary markers*”. And of course, there are interviewees who have dealt with enmity. M3 himself admits “*I had enemies—and still have—who wish me dead*”.

**Friendliness.** Another dimension of affability we considered is cordiality (Figure S1M), understood as the quality of being affectionate or maintaining kind relationships with others. This was explored with the following question: “*Were you someone who spoke kindly to others?*” As we can see, 96.5% answered “*very much*” or “*quite a lot*” regarding their cordiality (Figure S1L).

**Harmony.** When focusing on harmony, understood as a tendency towards consensus and avoiding conflict with others, we examined participants’ inclination to maintain adversarial relationships or experience conflict with their surroundings. It was found that 66.1% reported never having had enemies, and 97.4% stated they had had few or none at all (Figure S1N).

**Sensibility.** Another concept related to affability is sensitivity, or the ability to be moved by emotional stimuli and, by extension, to feel empathy for the problems of others. A total of 63.3% consider themselves to have a “*soft heart*” (Figure S1O).

**Sociability and Extroversion.** Our centenarians tend to be sociable, although not markedly so. About 55.5% of those interviewed always preferred to work in company rather than alone, but up to 33.9% preferred solitude, which reveals a noteworthy tendency toward individualism (Figure S1P). However, it should be noted that most of our centenarians worked in agriculture and thus had to perform tasks that could only be accomplished collectively. Collective work was accepted by 70.3% of the interviewees.

**Commensality.** A cultural factor linked to extroversion among Galician centenarians is the tendency to organise or participate in celebrations and banquets, or an affection for such events; this is what we call in Galicia being a “festive” person. In this regard, we considered the dimensions of commensality and festivity. The former refers to the enjoyment of organising and attending banquets with friends and family. We observed that 67% greatly or fairly enjoy commensality, and 71% enjoy festivities and consider themselves festive people (Figure S1Q).

**Festivity and individualism.** We can say that individualism is a trait frequently expressed by our centenarians (Figure S1R). The analysis of their narratives provides nuance to this observation. Most of the accounts reflect a day-to-day individualism centred around the home, family, and personal privacy. However, this individualistic tendency shifts in the context of community work or collective events such as festivals, celebrations, and funerals. Centenarians are often active participants in communal life and festivities (Figure S1S).

**Order and organization.** Another dimension of responsibility we explored is orderliness (the ability to place things where they belong, maintain cleanliness in their environment, pay attention to detail, and keep things orderly over time). A total of 92% of the interviewees consider themselves to have been orderly throughout their lives (Figure S1T). Another related dimension considered is organisation, understood as the ability to plan activities methodically through routines that minimize the need for improvisation, enable effective time management, and allow for distinguishing between what is urgent and what is important. Up to 80.5% consider themselves to be very or fairly organized (Figure S1U).

**Commitment.** Another dimension explored is commitment, understood as being disciplined in work habits, being capable of honouring agreements or commitments, and being someone others can rely on or trust. All respondents considered themselves to have been, throughout their lives, fairly or very reliable and committed individuals (Figure S1V).

**Industriousness.** This is expressed in our study as the tendency to dedicate a large portion of one’s time and energy to work, with the goal of achieving a sense of progress, productivity, or material growth. This trait is highly expressed in our study population, to the point of becoming one of their most outstanding characteristics. Regarding the intensity of work, 70.5% considered themselves to have worked a lot throughout their lives (Figure S1W), and 93.8% said that they have worked a lot or quite a lot (Figure S1X). As for the persistence of work, 99% acknowledged that they always had something to do, rarely had inactive days, and even fewer boring ones. This trait, like responsibility, is consistently confirmed by informants. However, in this case, the qualitative perspective is more compelling than the quantitative one. Industriousness emerges as the most reaffirmed quality on a biographical level. The interviewees frequently emphasize this aspect as central to their lives—whether as a source of pride (“*everything I achieved was through hard work*”), a path to prosperity (“*we escaped poverty thanks to our hard work*”), sometimes as a form of complaint (“*the only thing I did in life was work*”), or even as a life motto or piece of advice (“*everything comes from work*”).

**Personality.** Low emotional reactivity, as a dimension of stability, is a trait that our interviewees recognize in themselves. A cumulative 74% describe themselves as having a “*calm and relaxed*” temperament (Figure S2A). This trait has been cross-checked with the opinion of informants, when present, and verified through discourse analysis.

**Resilience.** Resilience is the capacity to adapt and to recover from adversity. Both participants and informants reported a “high” or “fairly high” level of adaptability in a combined total of 61.1% (Figure S2B). Another dimension of resilience we explored was adaptability to change. A total of 76.6% of interviewees adapted well to changes in circumstances, employment, housing, or life stage, regardless of whether those changes were positive or negative (Figure S2C). In cases where the changes were negative, interpreted as setbacks during life, 84% reported moving forward with ease or relative ease (Figure S2D).

**Life satisfaction.** Life satisfaction up to this point is closely related to resilience, as well as to the trait of emotional stability. A total of 96.2% of respondents said they were either “very” or “quite” satisfied with their lives so far (Figure S2E).

One dimension of stability is nervousness, understood as a state of hypervigilance toward the environment and a tendency to act impulsively. A total of 64.5% of participants deny having this trait (Figure S2F). There are gender differences (*p* = 0.01), with this trait being more commonly expressed in women.

Another aspect is irritability, approached as a tendency to remain frequently on edge or uncomfortable in the face of events, and to experience strong and often negative emotional reactions, such as anger or frustration, in response to everyday stimuli. A total of 70% of our centenarians describe themselves as little or not at all irritable (Figure S2G).

The trait of restlessness has been interpreted as a tendency to worry intensely about everyday matters, to experience anxiety in anticipation of events, and to exhibit a certain hypersensitivity to uncertainty. In this aspect, the majority of centenarians consider themselves to be “worriers”, with 67.0% describing themselves in this way (Figure S2H).

We explored the trait of serenity, defined as the experience of inner peace, emotional balance, and calm, as well as the possession of qualities such as patience and emotional self-control. This trait appears with high or moderate intensity in 71.7% of interviewees (Figure S2I).

Another trait examined is joyfulness, which is universally understood as a pleasant emotion expressed through visible signs such as laughter, joking, or positive words and actions toward oneself and others. A total of 83.2% of interviewees consider themselves joyful (Figure S2J).

**Spirituality.** In this study, we differentiate between religiosity and devotion. The former refers to participation in religious activities, often more traditional than belief-driven. The latter refers to a personal and emotional sense of connection or relationship with God or a divine equivalent. Regarding religiosity, 84% describe themselves as very or fairly religious (Figure S2K). As for devotion, 71% report communicating a lot or fairly often with God or divine entities to which they are devoted (Figure S2L). This communication typically involves asking for favours or expressing gratitude for favourable events. Interestingly, qualitative analysis reveals a predominant belief that there is no afterlife and that death marks a definitive end. For instance, M8, who was close to priests, says “*Anyone who believes there’s something beyond is mistaken. You die, and that’s it*.” Similarly, M3 states the following: “*After death, you’re dead—and that’s the end*.” Quantitatively, 53% of interviewees deny the existence of life after death.

It is very significant that we found a statistically significant relationship between gender and religiosity (*p* = 0.001), as well as between gender and devotion (*p* = 0.005). Both characteristics are more strongly expressed in women. The qualitative analysis shows that, in male discourse, invoking God is less frequent because it is interpreted as a sign of weakness, something that does not occur in female discourse. However, we did not find any association between spirituality and level of education or other traditional beliefs (witches, etc.).

Spirituality is a notable trait in our centenarian population. Nevertheless, this characteristic is more rooted in tradition (religiosity) than in a genuine personal feeling (devotion) or in the belief in an afterlife (transcendentalism). Moreover, this belief system is typical of a generation that received a religious (confessional) education.

**Sexuality and marital life**. Most participants reported having a happy relationship. In total, 80.6% indicated that their married life was very or quite happy (Figure S2M). Obviously, this excludes those who were single, who make up 10.4% of the sample. However, marital sexual activity was not intense. Only 40.3% of respondents stated that there was “a lot” or “quite a bit” of sex in their marriage (Figure S2N). In total, 21.7% said there was no sex at all. This figure may be skewed by the fact that the question was preceded by a statement respecting the respondent’s privacy, inviting them not to answer. As a result, 33% of participants chose not to respond to this question. Discourse analysis reveals that sex was not a priority in marital life. Possibly, the sinful or unhealthy connotations attributed to sexuality played a role, as reflected in the words of MRS, a single woman from Ourense, who said the following: “*I have always lived a healthy life; I have never had sexual relations with anyone*”. It is noteworthy that a statistically significant association was found between the amount of sexual activity and gender (*p* = 0.001). Men tend to choose the options “a lot” or “quite a bit”, while women tend to answer “little” or “none”. Discourse analysis helps explain this gender difference. Men are less hesitant to acknowledge their sexual activity and even tend to exaggerate it, while women tend to omit or downplay that aspect of their lives. Another generation-dependent observation is that men are less reluctant to admit extramarital sexual activity.

**Family and social support.** We adapted certain concepts to fit the understanding and idiosyncrasies of our interviewees. Using information from their own narratives and discourse, we identified five key aspects of family and social support that were of concern to our centenarians. These are defined as confidential, emotional, recreational, material, and affective support.

Confidential support, understood as the availability of someone with whom to talk about intimate and personal matters, was reported by 77.1% of interviewees as having been available “a lot” or “quite a bit” throughout their lives (Figure S2O).

Material support, defined as the availability of help with practical or financial matters, especially when the person could not cope alone (e.g., due to illness), was reported as “a lot” or “quite a bit” by 83.8% of participants (Figure S2P).

Recreational support, referring to the availability of someone with whom to share pleasant moments, leisure time, or even sexual companionship, was reported as widely available by 87.4% (Figure S2Q).

Emotional support, broadly defined as the presence of someone who cares about the person, overlaps with general social support. A total of 90.4% reported having this type of support “a lot” or “quite a bit” (Figure S2R).

Affective support, seen as deep emotional closeness typically provided by a partner or close family member—someone to love and be loved by—was reported as “a lot” or “quite a bit” by 88.5% of participants (Figure S2S).

In general, we can conclude that family and social support among the centenarians in our study has been, and continues to be, high. Most of them lived in rural or semi-urban settings, where social networks tend to be smaller and closer, allowing for stronger emotional and material bonds than in urban environments. Furthermore, close living arrangements with extended family members create a broad kinship network that helps ensure continued support.

**Level of education and its relationship to cognitive decline.** There is a clear and statistically significant relationship between educational level and cognitive decline, which was less common among those with more than primary education (*p* = 0.02), with a contingency coefficient of 0.28. Centenarians who had completed only primary education had an 88% higher risk of developing dementia (OR = 0.12).

**Education Level and Its Relationship with Dementia.** There is a statistically significant relationship between education level and dementia, with a lower incidence among those who attained education beyond primary school (*p* = 0.02), and a contingency coefficient of 0.28. The risk of dementia is 88% higher among centenarians who only completed primary education (OR = 0.12), (Figure S3A).

**Early Socioeconomic Status and Its Relationship with Dementia.** There is a higher incidence of dementia among those centenarians whose parents had no occupational qualifications beyond agricultural labour. However, this relationship is not statistically significant (*p* = 0.35), as shown in Figure S3B.

When we analysed the economic status of the parents in three categories (lower, similar, or higher than average) in relation to the presence or absence of dementia, we found no statistically significant relationship. However, when we grouped these categories into two (higher than average and not higher than average), a statistically significant relationship emerged (*p* = 0.026). In the binary regression model, we obtained an odds ratio of 0.40. That is, centenarians who had an above-average economic status during childhood had a 60% lower probability of developing dementia compared to those with average or below-average economic conditions. This finding is consistent with the existing literature. It is important to note that most centenarians lived in economically precarious environments. Precariousness was the norm, so having an average economic status did not mean being free from hardship. Only those who had a higher-than-average economic status escaped this condition. This situation brought several advantages regarding cognitive health and the development of cognitive reserve during childhood—such as better nutrition, greater access to proteins, and fewer vitamin deficiencies. Additionally, a better economic situation often made access to education possible. Although these centenarians had enough longevity factors to live to 100 years of age, the useful life of their brains did not always last as long. We therefore observe a statistically significant relationship between a higher early-life economic status and the absence of dementia (*p* = 0.026), as represented in Figure S2.

### 3.5. Relationship Between Each Variable and Cognitive Impairment

**Diet and Its Relationship with Dementia.** We did not find a statistical association between the presence or absence of cognitive impairment and dietary variables. There was no association with caloric intake (*p* = 0.42), nor with the consumption of certain foods such as meat (*p* = 0.63), fish (*p* = 0.29), or vegetables (*p* = 0.42). We did find a paradoxical association between sugar consumption and the absence of dementia (Table 2) (*p* = 0.03), although the association was weak (contingency coefficient 0.27).

This finding, which lacks biological plausibility, may instead reflect that access to sugars—especially in the first third of our centenarians’ lives—serves as a good indicator of the family’s socioeconomic status, which, as we have seen, is related to the onset of dementia.

**Water consumption and its relationship with dementia.** In our centenarian sample, we found a statistically significant association between dementia and a lower tendency to drink water (*p* = 0.02) (Figure S3C). We found no association with the type of water (*p* = 0.43) or with the source of irrigation water (*p* = 0.61). When treating water consumption as a dichotomous variable (high vs. low water intake), the association remains (*p* = 0.02). A binary logistic regression for these two categories yields an odds ratio of 2.65 (1.19–5.18), indicating a significant effect size. This finding could partly be explained by a health-conscious behaviour: those more mindful of drinking water may have also been more concerned about their health, adopting healthier habits throughout their lives.

**Sleep patterns and their relationship with dementia.** We found no association between dementia and sleep quality (*p* = 0.81), nor with having had more or fewer problems with insomnia (*p* = 0.15). There was also no association with the use of sleep aids (*p* = 0.61), or the number of sleep hours (*p* = 0.23). Therefore, sleep patterns do not appear, in our study, to be a dimension that conditions the presence of dementia in centenarians.

**Extreme longevity and its relationship with other variables.** “*Extreme longevity*” individuals, aged 105 or older, were a very small proportion (11.3%, n = 13). We found no statistically significant association between extreme longevity and sex (Figure S3D). (*p* = 0.31); it is not more common among women than men. It also does not depend on marital status (*p* = 0.93), water consumption (*p* = 0.29), or early socioeconomic status (*p* = 0.63). There was insufficient data to assess the associations with parental longevity or education level. Sleep quality also showed no association (*p* = 0.12).

**Alcohol consumption and its relationship with dementia.** We did not find any association between dementia and wine consumption (*p* = 0.36), nor by wine type (red or white) (*p* = 0.33) (Figure S3E), nor with the consumption of distilled spirits (*p* = 0.78). This may be because wine consumers are overwhelmingly moderate drinkers, and very few regularly consumed distilled beverages.

**Dependency and its relationship with other variables.** We treated the dependency variable as dichotomous, distinguishing between non-dependent centenarians and those with moderate or severe dependency. As expected, there is a statistically significant relationship between dementia and dependency. We also found a relationship with self-reported health in the past 12 months (*p* = 0.02).

Regarding dietary variables, only milk intake was significantly associated with dependency (*p* = 0.03), likely related to higher calcium intake and possibly better resistance to fractures. The most relevant association is between dementia and dependency, with a contingency coefficient of 0.41, indicating a moderate association, as shown in the bar chart. In our study, the odds ratio for the dementia–dependency relationship is 1.22.

**Gender differences regarding the studied variables.** Alcohol and wine consumption is associated with male sex. For wine (*p* = 0.04), the contingency coefficient is 0.32, and for distilled spirits (*p* = 0.01), it is 0.34. The contingency table for wine consumption shows that 12% of men drank a lot of wine, compared to just 1.2% of women. Tobacco use is also associated with male sex (*p* = 0.0001), as only one woman in the sample had been a smoker.

Some sleep-related variables also show gender differences, such as the use of sleep aids (*p* = 0.01) and the habit of taking naps (*p* = 0.01). Regarding sleep aids, only 6.7% of men had ever used them, compared to 36.5% of women—consistent with the literature indicating higher psychotropic drug use among women (Figure S3F).

Napping was more common among men; 46.7% of men reported frequent napping, compared to only 22.6% of women (Figure S3G). This may be due to women having a greater workload, at least in terms of time spent, leaving less time for leisure after meals due to kitchen duties and childcare. Male activities, on the other hand, were perceived as more intense and deserving of rest.

Dementia is also associated with sex among centenarians, as is known in the general population. In our study, 58.6% of men were free of dementia, compared to 35.7% of women (Figure S3H).

**Life assessment.** We define life assessment as the general evaluation of life at a given moment. We are interested in this evaluation at the very end of the life span. To assess this, we asked about life achievements (whether they attained the important things in life), overall life satisfaction, and what they would change if they could go back in time. Notably, 82% of centenarians said they had achieved the important things in life to a great or considerable extent (Figure S3I). The qualitative analysis provides insight into what these achievements are, which are mostly limited to the first three levels of Maslow’s hierarchy of needs: survival, safety, and belonging. Examples include F21: “*Work, eat, earn a little and pay in order to get the pension.*” F23: “*To have things well managed, have enough, and ensure the land doesn’t lie fallow, enough cabbage for the animals…*” M7: “*To have a family that doesn’t lack anything, everyone healthy, and getting along.*” Given these modest expectations—which rarely extend to esteem or self-actualization—they were largely fulfilled. This is reflected in the fact that 96.2% of centenarians expressed satisfaction with their lives.

**Life satisfaction. Are you satisfied with your life so far?** In general, they would not change much about their lives, even if they could (Figure S3I). In their narratives, the few things they would change mostly related to family: having children, not being separated from them during emigration, or buying a home and choosing a different place to live (closer to their children).

Centenarians are aware of information about healthy habits and popular ideas about longevity, such as calm, healthy diet, genetics, and care, but at heart, they are the first to be surprised by their own long lives. In their bewilderment, they attribute it to chance or divine intervention. However, there is not the same level of optimism regarding the experience of being a centenarian itself. In that case, the predominant discourse is negative and against extreme longevity.

## 4. Conclusions

This is the first mixed-methods study (quantitative and qualitative) on centenarians from Ourense. The findings highlight the central role of nutrition and lifestyle in achieving exceptional longevity. Participants’ diets were rooted in self-sufficiency, seasonal fresh produce, and community-based food sharing—reflecting a naturally balanced and sustainable approach to eating.

The Southern European Atlantic Diet (rich in local greens such as turnip tops and cabbage) played a central role. Most food was either homegrown or exchanged among neighbours, ensuring high nutrient density and minimal processing. Despite limited access to fish, the modest consumption of oily species such as sardines provided essential omega-3 fatty acids. Pork was a dietary mainstay due to tradition and availability, while milk was a staple and cheese considered a luxury. Diets were low in added sugars and salt, with honey and occasional homemade desserts serving as the primary sources of sweetness. These dietary practices supported cardiovascular and metabolic health, helped maintain a healthy body weight, and preserved cultural identity.

Beyond nutrition, psychosocial factors were also notable. Centenarians reported high levels of functional independence and satisfactory emotional well-being, supported by strong social and family networks, which are typical in rural and semi-urban settings. Although some episodic memory decline was observed, social connection remained intact in many participants.

In terms of personality, common traits included responsibility, industriousness, calmness, resilience in facing life’s challenges, and sociability. A tendency towards everyday individualism was observed, balanced by strong participation in communal events. Spirituality was widespread, although expressed more as a cultural tradition than as a transcendental belief system.

Regarding lifestyle, physical activity was embedded in daily routines rather than undertaken as structured exercise. Self-maintained gardens—present in 96% of participants—not only ensured food quality but also promoted regular physical movement. This integration of diet and lifestyle reflects a nutritionally rich and environmentally conscious model from which modern populations might learn. Sleep patterns were varied, with less reliance on naps and hypnotic medications than typically assumed among older adults. The prevalence of insomnia was comparable to that of the general population. Other possible contributing factors included low tobacco use and physically active work lives.

Finally, in our study, and following scientific literature, different combinations of variables (lifestyle, diet, physical exercise, environmental and social factors, personality, etc.) result in a group of people with extraordinary longevity compared to their peers, with some of them in good health (optimal ageing). These findings support the growing emphasis on modifiable environmental and lifestyle factors as critical components of healthy ageing. Anyway, genetic analyses of this sample could complement the information needed to shed more light on the phenomenon of extreme longevity in humans.

## Figures and Tables

**Table 1:** Comparison of the most notable nutritional aspects between the regions of Okinawa, Sardinia and Ourense.

Food Item	Ourense (303,450 Inhabitants)	Sardinia (56,000 Inhabitants)	Okinawa (1,285,000 Inhabitants)
Pork	Most consumed meat	Most consumed meat	Most consumed meat. All parts are eaten, including tendons and cartilage
Cheese	Yes, with quality labels. Cow	Yes, widely consumed. Sheep and goat	Little. Cow
Vegetables	Mainly brassica varieties	Smaller quantities. Diet predominantly animal-based	Yes, combined with other foods
Preferred fish type	Oily fish from the Atlantic Ocean	White fish from the Mediterranean Sea	Freshwater fish
Milk	Significant quantity. Cow	Significant, rich in arzanol	Significant
Wine	Very little	Moderate, consumed with meals	Little
Typical dish	Galician stew	Minestrone with onion, carrots, legumes, and fennel	Food combination: fish with pig liver, garlic, and carrot
Seaweed	Not consumed	Rarely consumed	Frequently consumed (Saccharina japonica)
Bread	Yes, with Protected Geographical Indication	Yes, served with meals. Very distinctive	No
Caloric restriction	No (2200–2700 kcal)	No (2200–2700 kcal)	Yes (1800–2200 kcal)
Cured meats	*Chorizo*	Plant-complemented cured meats. “Guanciale”	Rarely consumed
Type of fat	Extra virgin olive oil	Lard and pork fat until nutritional transition	Pork fat
Condiment	Paprika	Saba (grape syrup)	Turmeric
Other characteristic foods	Chestnuts	Mastic tree oil	Soya and tofu

**Table 2:** Relationship between sugar consumption and the presence of dementia.

Sugars	A Lot	Quite a Bit	A Little	None	Total
No Dementia	10	11	25	1	47
Dementia	4	9	50	1	64
Total	14	20	75	2	111

## Data Availability

The data presented in this study are not publicly available due to ethical restrictions. The study was approved by an ethics committee and all data were anonymised for publication.

## Supplementary Materials

The following supporting information can be downloaded at: https://www.mdpi.com/article/10.3390/nu17132231/s1, Section S1: Table S1: Variables and dimensions. Table S2: Thematic coding framework based on the main discourse categories identified in centenarians’ interviews. Section S2: Information Sheet for Adult Participants. Section S3: Consent Form for Participation in a Research Study. Section S4: Questionnaire. Section S5: Table S3: Pfeiffer Test. Table S4: Barthel Test. Figure S1: Assessment of physical activity, sleep, and other health habits of centenarians. Figure S2: Assessment of emotional factors in centenarians. Figure S3: Study of factors influencing dementia and other aspects affecting longevity.

## Abbreviations

The following abbreviations are used in this manuscript:

BMI	Body Mass Index
CI	Centenarian Index
CVD	Cardiovascular Disease
DASH	Dietary Approaches to Stop Hypertension
LI	Longevity Index
M/F	Male/Female
NECS	New England Centenarian Study
PSS	Perceived Stress Scale
PUFA	Polyunsaturated Fatty Acids
RAS	Relationship Assessment Scale
SEAD	Southern European Atlantic Diet
SPSS	Statistical Package for Social Sciences
SWLS	Satisfaction with Life Scale
UPF	Ultra-Processed Food
GPAQ	Global Physical Activity Questionnaire

## References

[1] Dakic T., Jevdjovic T., Vujovic P., Mladenovic A. (2022). The Less We Eat, the Longer We Live: Can Caloric Restriction Help Us Become Centenarians?. Int. J. Mol. Sci..

[2] Sebastiani P., Perls T.T. (2012). The Genetics of Extreme Longevity: Lessons from the New England Centenarian Study. Front. Genet..

[3] Boccardi M., Boccardi V. (2019). Psychological Wellbeing and Healthy Aging: Focus on Telomeres. Geriatrics.

[4] Ljungquist B., Berg S., Lanke J., McClearn G.E., Pedersen N.L. (1998). The Effect of Genetic Factors for Longevity: A Comparison of Identical and Fraternal Twins in the Swedish Twin Registry. J. Gerontol. A Biol. Sci. Med. Sci..

[5] Royston K.J., Tollefsbol T.O. (2015). The Epigenetic Impact of Cruciferous Vegetables on Cancer Prevention. Curr. Pharmacol. Rep..

[6] Brooks-Wilson A.R. (2013). Genetics of Healthy Aging and Longevity. Hum. Genet..

[7] Komaki S., Nagata M., Arai E., Otomo R., Ono K., Abe Y., Ohmomo H., Umekage S., Shinozaki N.O., Hachiya T. (2023). Epigenetic Profile of Japanese Supercentenarians: A Cross-Sectional Study. Lancet Healthy Longev..

[8] Coppedè F., Franzago M., Giardina E., Nigro C.L., Matullo G., Moltrasio C., Nacmias B., Pileggi S., Sirchia S.M., Stoccoro A. (2022). A Perspective on Diet, Epigenetics and Complex Diseases: Where Is the Field Headed Next?. Epigenomics.

[9] Morris B.J., Willcox B.J., Donlon T.A. (2019). Genetic and Epigenetic Regulation of Human Aging and Longevity. Biochim. Biophys. Acta BBA—Mol. Basis Dis..

[10] Yu Z., Zhang F., Xu C., Wang Y. (2022). Association between Circulating Antioxidants and Longevity: Insight from Mendelian Randomization Study. BioMed Res. Int..

[11] Elkashty O.A., Tran S.D. (2021). Sulforaphane as a Promising Natural Molecule for Cancer Prevention and Treatment. Curr. Med. Sci..

[12] Bernkopf D.B., Daum G., Brückner M., Behrens J. (2018). Sulforaphane Inhibits Growth and Blocks Wnt/β-Catenin Signaling of Colorectal Cancer Cells. Oncotarget.

[13] Zhao G., He F., Wu C., Li P., Li N., Deng J., Zhu G., Ren W., Peng Y. (2018). Betaine in Inflammation: Mechanistic Aspects and Applications. Front. Immunol..

[14] Feinberg A.P., Levchenko A. (2023). Epigenetics as a Mediator of Plasticity in Cancer. Science.

[15] El-Seweidy M.M., Ali S.I., Elsweify S.E., Ali A.A., Mashhour M.M. (2017). Omega3 Fatty Acids Intake Versus Diclofenac in Osteoarthritis Induced in Experimental Rats. Funct. Foods Health Dis..

[16] Bowen K.J., Harris W.S., Kris-Etherton P.M. (2016). Omega-3 Fatty Acids and Cardiovascular Disease: Are There Benefits?. Curr. Treat. Options Cardiovasc. Med..

[17] Biagi E., Franceschi C., Rampelli S., Severgnini M., Ostan R., Turroni S., Consolandi C., Quercia S., Scurti M., Monti D. (2016). Gut Microbiota and Extreme Longevity. Curr. Biol..

[18] Tafaro L., Cicconetti P., Baratta A., Brukner N., Ettorre E., Marigliano V., Cacciafesta M. (2007). Sleep Quality of Centenarians: Cognitive and Survival Implications. Arch. Gerontol. Geriatr..

[19] Aiello A., Accardi G., Ali S., Caruso C., Chen M., Vivo I., Ligotti M., Scapagnini G., Davinelli S., Candore G. (2021). Possible Association of Telomere Length with Sleep Duration. A Preliminary Pilot Study in a Sicilian Cohort with Centenarians. Transl. Med. UniSa.

[20] Demakakos P., Nazroo J., Breeze E., Marmot M. (2008). Socioeconomic Status and Health: The Role of Subjective Social Status. Soc. Sci. Med..

[21] Packard C.J., Bezlyak V., McLean J.S., Batty G.D., Ford I., Burns H., Cavanagh J., Deans K.A., Henderson M., McGinty A. (2011). Early Life Socioeconomic Adversity Is Associated in Adult Life with Chronic Inflammation, Carotid Atherosclerosis, Poorer Lung Function and Decreased Cognitive Performance: A Cross-Sectional, Population-Based Study. BMC Public Health.

[22] Zimmer Z., Jagger C., Chiu C.-T., Ofstedal M.B., Rojo F., Saito Y. (2016). Spirituality, Religiosity, Aging and Health in Global Perspective: A Review. SSM—Popul. Health.

[23] Tafaro L., Tombolillo M.T., Brükner N., Troisi G., Cicconetti P., Motta M., Cardillo E., Bennati E., Marigliano V. (2009). Stress in Centenarians. Arch. Gerontol. Geriatr..

[24] Aliberti S.M., De Caro F., Funk R.H.W., Schiavo L., Gonnella J., Boccia G., Capunzo M. (2022). Extreme Longevity: Analysis of the Direct or Indirect Influence of Environmental Factors on Old, Nonagenarians, and Centenarians in Cilento, Italy. Int. J. Environ. Res. Public. Health.

[25] Buettner D., Skemp S. (2016). Blue Zones: Lessons from the World’s Longest Lived. Am. J. Lifestyle Med..

[26] Willcox B.J., Willcox D.C., Todoriki H., Fujiyoshi A., Yano K., He Q., Curb J.D., Suzuki M. (2007). Caloric Restriction, the Traditional Okinawan Diet, and Healthy Aging. Ann. N. Y. Acad. Sci..

[27] Garagnani P., Bacalini M.G., Pirazzini C., Gori D., Giuliani C., Mari D., Di Blasio A.M., Gentilini D., Vitale G., Collino S. (2012). Methylation of ELOVL2 Gene as a New Epigenetic Marker of Age. Aging Cell.

[28] Ministerio de Agricultura, Pesca y Alimentación Informe Del Consumo Alimentario en España 2022. https://www.mapa.gob.es/eu/alimentacion/temas/consumo-tendencias/informe-consumo-2022-baja-res_tcm35-655390.pdf.

[29] Mendoza K., Smith-Warner S.A., Rossato S.L., Khandpur N., Manson J.E., Qi L., Rimm E.B., Mukamal K.J., Willett W.C., Wang M. (2024). Ultra-Processed Foods and Cardiovascular Disease: Analysis of Three Large US Prospective Cohorts and a Systematic Review and Meta-Analysis of Prospective Cohort Studies. Lancet Reg. Health—Am..

[30] Kliemann N., Al Nahas A., Vamos E.P., Touvier M., Kesse-Guyot E., Gunter M.J., Millett C., Huybrechts I. (2022). Ultra-Processed Foods and Cancer Risk: From Global Food Systems to Individual Exposures and Mechanisms. Br. J. Cancer.

[31] Vivanco P.G., Taboada P., Coelho A. (2023). The Southern European Atlantic Diet and Its Supplements: The Chemical Bases of Its Anticancer Properties. Nutrients.

[32] Instituto Universitario de Ciencias de la Salud Universidad de A Coruña Encuesta Sobre Los Hábitos Alimentarios de La Población Adulta Gallega. https://www.sergas.es/cas/Publicaciones/Docs/SaludPublica/PDF-2153-es.pdf.

[33] Pes G.M., Tolu F., Dore M.P., Sechi G.P., Errigo A., Canelada A., Poulain M. (2015). Male Longevity in Sardinia, a Review of Historical Sources Supporting a Causal Link with Dietary Factors. Eur. J. Clin. Nutr..

[34] Guallar-Castillón P., Oliveira A., Lopes C., López-García E., Rodríguez-Artalejo F. (2013). The Southern European Atlantic Diet Is Associated with Lower Concentrations of Markers of Coronary Risk. Atherosclerosis.

[35] GALIAT Galicia Alimentación Atlántica DIETA ATLÁNTICA GALLEGA Productos Autóctonos Beneficiosos Para La Salud. https://galiat6mas7.com/es/categorias.php?var1=Proyecto&nar1=820&var2=Introducci%F3n&nar2=820&vez=1&metatitle=.

[36] Calvo-Malvar M., Benítez-Estévez A.J., Sánchez-Castro J., Leis R., Gude F. (2021). Effects of a Community-Based Behavioral Intervention with a Traditional Atlantic Diet on Cardiometabolic Risk Markers: A Cluster Randomized Controlled Trial (“The GALIAT Study”). Nutrients.

[37] Carballo-Casla A., Ortolá R., García-Esquinas E., Oliveira A., Sotos-Prieto M., Lopes C., Lopez-Garcia E., Rodríguez-Artalejo F. (2021). The Southern European Atlantic Diet and All-Cause Mortality in Older Adults. BMC Med..

[38] Govindaraju D., Atzmon G., Barzilai N. (2015). Genetics, Lifestyle and Longevity: Lessons from Centenarians. Appl. Transl. Genomics.

[39] (2022). Instituto Nacional de Estadística Número de Personas Centenarias En Ourense y Lugo.Ministerio de Economía y Hacienda. https://www.ine.es/jaxi/Datos.htm?path=/t20/e245/p04/provi/l0/&file=0ccaa003.px#.

[40] Pfeiffer E. (1975). A Short Portable Mental Status Questionnaire for the Assessment of Organic Brain Deficit in Elderly Patients. J. Am. Geriatr. Soc..

[41] Mahoney F.I., Barthel D.W. (1965). Functional Evaluation: The Barthel Index. Md. State Med. J..

[42] Harris K.M., Gaffey A.E., Schwartz J.E., Krantz D.S., Burg M.M. (2023). The Perceived Stress Scale as a Measure of Stress: Decomposing Score Variance in Longitudinal Behavioral Medicine Studies. Ann. Behav. Med..

[43] Hendrick S.S. (1988). A generic measure of relationship satisfaction. J. Marriage Fam..

[44] Lv Y., Mao C., Yin Z., Li F., Wu X., Shi X. (2019). Healthy Ageing and Biomarkers Cohort Study (HABCS): A Cohort Profile. BMJ Open.

[45] Collerton J., Barrass K., Bond J., Eccles M., Jagger C., James O., Martin-Ruiz C., Robinson L., Von Zglinicki T., Kirkwood T. (2007). The Newcastle 85+ Study: Biological, Clinical and Psychosocial Factors Associated with Healthy Ageing: Study Protocol. BMC Geriatr..

[46] Rafiei H., Ashrafizadeh M., Ahmadi Z. (2020). MicroRNAs as Novel Targets of Sulforaphane in Cancer Therapy: The Beginning of a New Tale?. Phytother. Res..

[47] Ministerio de Agricultura, Pesca y Alimentación Encuesta Sobre Superficies y Rendimientos Cultivos (ESYRCE). https://www.mapa.gob.es/dam/mapa/contenido/estadisticas/temas/estadisticas-agrarias/2.agricultura/1.-encuesta-sobre-superficies-y-rendimientos-de-cultivos--esyrce/2023/boletin20231.pdf.

[48] Asociación Ourensividad Estudio Del Fenómeno de Los Centenarios de Ourense. https://ourensividad.com/ourense-clave-para-la-longevidad-el-fenomeno-de-los-centenarios/.

[49] Filippou C.D., Tsioufis C.P., Thomopoulos C.G., Mihas C.C., Dimitriadis K.S., Sotiropoulou L.I., Chrysochoou C.A., Nihoyannopoulos P.I., Tousoulis D.M. (2020). Dietary Approaches to Stop Hypertension (DASH) Diet and Blood Pressure Reduction in Adults with and without Hypertension: A Systematic Review and Meta-Analysis of Randomized Controlled Trials. Adv. Nutr..

[50] Willcox D.C., Willcox B.J., Todoriki H., Suzuki M. (2009). The Okinawan Diet: Health Implications of a Low-Calorie, Nutrient-Dense, Antioxidant-Rich Dietary Pattern Low in Glycemic Load. J. Am. Coll. Nutr..

[51] Pes G.M., Dore M.P., Tsofliou F., Poulain M. (2022). Diet and Longevity in the Blue Zones: A Set-and-Forget Issue?. Maturitas.

[52] Fastame M.C. (2022). Well-Being, Food Habits, and Lifestyle for Longevity. Preliminary Evidence from the Sardinian Centenarians and Long-Lived People of the Blue Zone. Psychol. Health Med..

[53] Agabio R., Pisanu C., Minerba L., Gessa G.L., Franconi F. (2021). Gender Differences among Sardinians with Alcohol Use Disorder. J. Clin. Med..

