# Supplementary material for: Exploring the Dietary Patterns and Health Behaviours of Centenarians in Ourense (Spain): Adherence to the Southern European Atlantic Diet
Source: Nutrients. 2025 Jul 5;17(13):2231. doi: 10.3390/nu17132231 (PMC12252380; doi:10.3390/nu17132231)

Supplementary Material

Exploring the Dietary Patterns and Health Behaviours of Centenarians in Ourense (Spain): Adherence to the Southern European Atlantic Diet

Pablo García-Vivanco[1,2*], Roberto Fernandez[3], Rosa Meijide-Faílde, [4,5] Esperanza Navarro-Pardo[6] Cristina Conde[7], Ricardo de la Fuente[8], Cristina Margusinos[9], Alberto Rodríguez[10], Ana Canelada[11], Pablo Taboada[12, 13], Alberto Cepeda[14] and Alberto Coelho[12, 13, 15*]

1 Spanish Academy of Nutrition and Dietetics, 31006 Pamplona, Spain
2 Nutrition and Digestive Working Group, Spanish Society of Clinical, Family, and Community Pharmacy (SEFAC), 28045 Madrid, Spain
3 Allariz Health Center, Galician Health Service (SERGAS), San Rosendo Foundation, 32660 Ourense, Spain
4 Cell Therapy and Regenerative Medicine Research Group, Institute of Biomedical Research of La Coruña (INIBIC), Interdisciplinary Centre for Chemistry and Biology (CICA), University of A Coruña, 15071 A Coruña, Spain
5 Department of Physiotherapy, Medicine and Biomedical Sciences, University of A Coruña, 15071 A Coruña, Spain
6 Department of Developmental and Educational Psychology, University of Valencia, 46010 Valencia, Spain
7 Official College of Pharmacists of Ourense, 32004 Ourense, Spain
8 Official College of Dietitians-Nutritionists of Galicia, 15190 A Coruña, Spain
9 Toén Health Center, Galician Health Service (SERGAS), 32930 Toén, Spain
10 Verín Health Center, Galician Health Service (SERGAS), 32600 Verín, Spain
11 Department of Health Sciences, University of Málaga, 29016 Málaga, Spain
12 Institute of Materials-USC (IMATUS), University of Santiago de Compostela, 15782 Santiago de Compostela, Spain
13 Colloids and Polymers Physics Group, Department of Physics of Particles, Faculty of Physics, University of Santiago de Compostela, 15782 Santiago de Compostela, Spain
14 Department of Analytical Chemistry, Nutrition and Food Science, University of Santiago de Compostela, 15782 Santiago de Compostela, Spain
15 Department of Organic Chemistry, Faculty of Pharmacy, University of Santiago de Compostela, 15782 Santiago de Compostela, Spain
* Correspondence: pablogvivanco@gmail.com (P.G.-V.); albertojose.coelho@usc.es (A.C.)

Table of contents:

Section S1……………………………………………………………………………………………S2
Table S1: Variables and dimensions……………………………………………………………S2
Table S2: Thematic coding framework based on the main discourse categories identified in centenarians' interviews……………………………………………………………S8
Section S2: Information Sheet for Adult Participants…………………………………………S9
Section S3: Consent Form for Participation in a Research Study……………………………S10
Section S4: Questionnaire…………………………………………………………………………S12
Section S5……………………………………………………………………………………………S18

Table S3: Pfeiffer Test……………………………………………………………………………S18
Table S4: Barthel Test……………………………………………………………………………S19
Figure S1……………………………………………………………………………………………...S20
Figure S2……………………………………………………………………………………………...S21
Figure S3……………………………………………………………………………………………...S22

Section S1

Table S1: Variables and dimensions

DIMENSION	VARIABLE	VALUES	DEFINITION
LONGEVITY	Centenarian index	Numerical	Ratio between the number of centenarians and the number of people over 90 years old in the municipality
	Longevity index	Numerical	Ratio between the number of centenarians and the number of people over 65 years old in the municipality
SOCIODEMOGRAPHIC	Age	Numerical	Age
	Sex	M/F	Sex
	Residence	Rural/Urban	Type of area where the person lives
	Marital status	Married/Single/Widowed/Separated	Marital status during most of adult life
	Number of children	Numerical	Number of children
ENVIRONMENTAL	Altitude	Low (0–300 m), Medium (300–600 m), High (>600 m)	Altitude of residence
	Mean temperature	Numerical	Annual average temperature in municipality
	Mean precipitation	Numerical	Annual average precipitation in municipality
	Radon exposure	Zone I, II, III	Level of radon exposure at main residence
	Thermal area	Yes/No	Whether main residence is in a thermal area
HEALTHY CENTENARIANSHIP	Pfeiffer test	Numerical	Cognitive assessment test
	Barthel test	Numerical	Functional assessment test
FAMILY HISTORY	Mother's age at death	Numerical	Age at which mother died
	Father's age at death	Numerical	Age at which father died
	Siblings’ age at death	Average	Average age of siblings at death (excluding avoidable or unnatural deaths)

DIMENSION	VARIABLE	VALUES	DEFINITION
	Other centenarians	Numerical	Number of other centenarians in the family
HEALTH	Self-perceived health	Good/Fair/Poor	Perceived general health
	Number of medications	Numerical	Indirect indicator of objective health
	Pain scale (VAS)	Numerical (0–10)	Visual analog scale for physical pain
DIET	Food amount	Much/Fair/Little/None	Quantity of food intake compared to peers
	Number of meals	Numerical	Number of meals per day
	Carbohydrates	Bread/Potatoes/Pasta/Others	Quantity of carbohydrate intake compared to peers
	Meat amount	Much/Normal/Little	Quantity of meat consumed compared to peers
	Type of meat	Red/White	Predominant type of meat in diet
	Fish amount	Much/Normal/Little	Quantity of fish consumed compared to peers
	Type of fish	Oily/White	Type of fish most commonly consumed
	Fruit amount	Much/Normal/Little	Quantity of fruit consumed compared to peers
	Legumes	Much/Normal/Little/None	Quantity of legumes consumed compared to peers
	Vegetables	Much/Normal/Little/None	Quantity of vegetables consumed compared to peers
	Type of vegetables	Much/Normal/Little/None	
	Turnip greens	Much/Normal/Little/None	Quantity of turnip greens consumed compared to peers
	Other vegetables	Much/Normal/Little/None	Quantity of other vegetables consumed
	Homegrown vs. store-bought	Much/Normal/Little/None	If most vegetables were homegrown or purchased
	Sugars	Much/Normal/Little/None	Sugar intake compared to others
	Salt	Much/Normal/Little/None	Salt intake compared to others
	Milk	Much/Normal/Little/None	Milk intake compared to others
	Cheese	Much/Normal/Little/None	Cheese intake compared to others
	Wine	Much/Normal/Little/None	Wine intake compared to others

DIMENSION	VARIABLE	VALUES	DEFINITION
	Spirits	Much/Normal/ Little/None	Distilled alcohol intake compared to others
	Excluded foods	Open-ended	Foods the person never consumed
	Weight status	Overweight/ Normal/Thin	Usual weight status during adult life
**WATER**	Water type	Bottled/Piped/Well or Spring	Type of water most commonly consumed
	Water amount	Approx. number of glasses	Approximate daily water intake
	Irrigation water	Piped/Well	Type of water used for homegrown garden irrigation
**THERMALISM**	Thermal water use	Much/Fair/Little/ None	Use of thermal or medicinal water
	Visits per year	Numerical	Frequency of spa visits per year
	Visit duration	Days	Number of days per spa visit
	Spas visited	Open-ended	Names of spas visited
**PHYSICAL ACTIVITY**	Movement tendency	Much/Fair/Little/ None	Tendency to move or avoid sedentary lifestyle
	Activity level	Much/Fair/Little/ None	Physical activity level compared to peers
	Physical work	Much/Fair/Little/ None	Whether job involved physical effort
	Sports	Much/Fair/Little/ None	Whether person practiced sports regularly
**SLEEP**	Sleep satisfaction	Much/Fair/Little/ None	Sleep quality compared to peers
	Sleep problems	Much/Fair/Little/ None	Difficulty sleeping
	Sleeping pills	Much/Fair/Little/ None	Use of sleeping medication
	Tendency to sleep	Much/Fair/Little/ None	Whether they were a sleepy person
	Sleep duration	>7h / 5–7h / <5h	Average daily sleep duration
	Napping	Yes/Sometimes/ No	Whether they took naps
**INTELLECTUAL ACTIVITY**	Reading	Much/Fair/Little/ None	Reading habits compared to peers
	Education level	Primary/ Secondary/Higher	Education level achieved
	Intellectual ability	Much/Fair/Little/ None	Self-perceived academic competence
**PERCEIVED STRESS**	Sense of control	Much/Fair/Little/ None	Feeling of control over one's life
	Tension	Much/Fair/Little/ None	Frequency of feeling nervous

DIMENSION	VARIABLE	VALUES	DEFINITION
	Stressful events	Spouse death/Divorce/ Child death/ Imprisonment	Major life stressors (top four in Holmes and Rahe stress scale)
	Economic status	Much/Fair/Little/ None	Whether they had enough money to live comfortably
OCCUPATIONAL	Diligence	Much/Normal/ Little/None	How hardworking they were
	Motivation	Much/Normal/ Little/None	How busy they kept themselves
	Emigration	Yes/No	Whether they emigrated
	Time abroad	Numerical (years)	How long they lived abroad
	Destination	National/Continent al/Intercontinental	Destination of emigration
SOCIAL SUPPORT	Emotional support	Much/Fair/Little/ None	Had someone to talk to
	Material support	Much/Fair/Little/ None	Had someone to help when sick
	Intimacy	Much/Fair/Little/ None	Had someone to share concerns with
	Leisure	Much/Fair/Little/ None	Had someone to enjoy time with
	Affection – Love	Much/Fair/Little/ None	Felt loved
	Affection – Care	Much/Fair/Little/ None	Felt cared for
VITAL ACTIVITY	Stability 1	Much/Fair/Little/ None	Whether they got angry easily
	Stability 2	Much/Fair/Little/ None	Whether they were a cheerful person
	Stability 3	Much/Fair/Little/ None	Whether they were calm and relaxed
	Affability 1	Much/Fair/Little/ None	Got along with people
	Affability 2	Much/Fair/Little/ None	Spoke kindly to others
	Affability 3	Much/Fair/Little/ None	Was kind-hearted
	Conscientiou sness 1	Much/Fair/Little/ None	Was orderly
	Conscientiou sness 2	Much/Fair/Little/ None	Was methodical
	Conscientiou sness 3	Much/Fair/Little/ None	Was dutiful
	Curiosity 1	Much/Fair/Little/ None	Had lots of ideas
	Curiosity 2	Much/Fair/Little/ None	Tried new things

DIMENSION	VARIABLE	VALUES	DEFINITION
	Curiosity 3	Much/Fair/Little/ None	Took on new activities
	Curiosity 4	Much/Fair/Little/ None	Kept up with world affairs
	Extraversion 1	Much/Fair/Little/ None	Liked being around people
	Extraversion 2	Much/Fair/Little/ None	Liked talking to people
	Extraversion 3	Much/Fair/Little/ None	Made friends easily
	Extraversion 4	Much/Fair/Little/ None	Enjoyed parties
	Resilience 1	Much/Fair/Little/ None	Recovered quickly from distress
	Resilience 2	Much/Fair/Little/ None	Adapted easily to change
	Resilience 3	Much/Fair/Little/ None	Persisted after failure
	Resilience 4	Much/Fair/Little/ None	Saw the positive side of things
SPIRITUALITY	Inner peace	Much/Fair/Little/ None	Felt at peace with oneself
	Religious practice	Much/Fair/Little/ None	Was a devout person
	Belief	Much/Fair/Little/ None	Prayed to God
	Belief in afterlife	Much/Fair/Little/ None	Believed in life after death
SATISFACTION	Marital satisfaction	Much/Fair/Little/ None	Satisfaction with marriage
	Sexual satisfaction 1	Much/Fair/Little/ None	Satisfaction with sex life
	Sexual satisfaction 2	Much/Fair/Little/ None	Frequency of sexual activity
	Global satisfaction	Much/Fair/Little/ None	Satisfaction with life overall
	Retrospective change	Much/Fair/Little/ None	Willingness to change past
	Life achievement	Yes/No	Whether they accomplished important life goals

Table S2: Thematic coding framework based on the main discourse categories identified in centenarians' interviews

FIRST-ORDER CATEGORIES	SECOND-ORDER CATEGORIES	DEFINITION
**HEALTH**	Self-perceived health	Codes related to self-perception of health
**DIET**	Frugality	Codes related to satiety
	Carbohydrates	Codes concerning carbohydrate intake
	Meat	Statements about meat, quantities, types, and preferences
	Fish	Statements about fish, types, and preferences
	Fruit	Statements about fruit, types, and preferences
	Legumes	Statements about legumes, types, and preferences
	Vegetables	Statements about vegetables, types, and preferences
	Self-cultivation	Codes focused on home-growing food
	Sugars	Statements about sweets, types, and preferences
	Salt	Statements about salt, quantities, and beliefs
	Milk	Statements about milk and dairy products, types, and preferences
	Wine	Statements about wine and patterns of consumption
	Spirits	Statements about spirits and patterns of consumption
	Excluded foods	Codes concerning excluded foods
	Weight	Statements about body weight throughout life
**WATER**	Drinking water	Statements about water intake and consumption patterns
	Irrigation water	Statements about water used for irrigation
**THERMALISM**	Spas	Statements about spa use and frequency
	Thermalism	Statements about beliefs in the benefits of thermalism
**PHYSICAL ACTIVITY**	Activity integrated into daily life	Codes related to patterns of work-related physical activity
	Sport	Codes about sport practice and related beliefs

Section S2: Information Sheet for Adult Participants

STUDY TITLE: *The Phenomenon of Centenarians in Ourense: A Quantitative and Qualitative Study*
PRINCIPAL INVESTIGATOR: Roberto Fernández Álvarez

INSTITUTION: Healthcare Area of Ourense, Verín, and O Barco de Valdeorras
This document aims to provide you with information about a research study in which you are invited to participate. This study has been approved by the Research Ethics Committee of Pontevedra-Vigo-Ourense.
If you decide to participate, you will receive personalized information from the researcher. Please read this document carefully and ask any questions you may have to fully understand the details. You are welcome to take this document home, discuss it with others, and take the time you need before deciding whether or not to participate.
Participation is entirely voluntary. You may choose not to participate or withdraw your consent at any time without giving a reason. This decision will not affect your relationship with healthcare professionals or the healthcare services to which you are entitled.

What is the purpose of the study?
This study aims to identify lifestyle habits or personality traits that may be common among individuals aged 100 or older.

Why am I being invited to participate?
You are being invited to participate because you are 100 years old or older.

What does participation involve?
If you agree to participate, a member of the research team will conduct an interview with you covering your lifestyle and habits (sleep, diet, physical activity, stress, etc.). The interview will take place in person at a time that suits you, or by phone if an in-person meeting is not possible.
The interview will be audio-recorded for later analysis. The findings will be shared through scientific and/or general publications to help improve health care and well-being in others.
You may also be contacted again to provide additional data related to the study.

Where will the interview take place?
The interview will be conducted in your home. If you prefer another location, please let the research team know when they contact you.

How long will the interview last?

Approximately 60 minutes.

Are there any inconveniences or risks?
Your participation will not require travel or cause discomfort. You will be asked to recall and share personal past experiences related to your health.

Will I benefit from participating?
There is no direct benefit to you from participating. However, your contribution may help uncover useful insights for promoting longevity in others.

Will I receive the study results?
If you wish, a summary of the study findings can be provided to you.

Will the results be published?
Yes, the findings may be published in scientific journals. However, no information that could identify you will be shared.

What about data protection?
Your personal data will be collected, stored, processed, and shared in accordance with the General Data Protection Regulation (EU Regulation 2016/679) and Spanish data protection laws.
The institution conducting the study is the data controller. You may contact the Data Protection Officer at: **dpd@sergas.es**
Your data will be pseudonymized (coded) — only the research team will know the code that links your identity to the data.
You have the right to access, correct, delete, limit, or oppose the processing of your data. You may also request a copy of your data or ask for it to be transferred to another party (data portability).
To exercise these rights, contact the Data Protection Officer or the Principal Investigator at:
📧 **Roberto.Fernandez.Alvarez@sergas.es**
📞 **+34 646 383 132**
You also have the right to file a complaint with the Spanish Data Protection Agency if you believe your rights have not been respected.
Only the research team and authorized health authorities (bound by confidentiality) will have access to your full data. Anonymous data may be shared with third parties for research purposes. If data are transferred to countries outside the EU, equivalent data protection standards will apply.
At the end of the study or once the legal retention period has expired, your data will either be deleted or anonymized for future research, depending on the option you choose on the consent form.

Are there any financial interests involved in this study?
This study is funded by the *Ourensividad* Association in collaboration with the Ourense, Verín, and O Barco de Valdeorras Healthcare Area. The researcher receives no financial compensation. You will not be paid for participating.
If any commercial products or patents result from the study, you will not receive any economic benefit.

How can I contact the research team?

For any questions or further information, you may contact the Principal Investigator:

✉ **Roberto.Fernandez.Alvarez@sergas.es**

📞 **+34 646 383 132**

Thank you very much for your collaboration.

Section S3: Consent Form for Participation in a Research Study

Study Title: *The Phenomenon of Centenarians in Ourense*

I, ...

- Have read the Participant Information Sheet for the above-mentioned study, which was provided to me, and I had the opportunity to speak with: .... and to ask all questions I had about the study.
- I understand that my participation is voluntary and that I may withdraw from the study at any time, without having to give a reason and without this affecting my medical care in any way.
- I agree to the use of my data under the conditions outlined in the Participant Information Sheet.
- I freely give my consent to participate in this study.

Upon completion of this study, I agree that my data will be:

___Deleted

___Kept in anonymized form for future use in other research projects

Signed: Participant　　　　**Signed:** Researcher requesting consent

Full Name:　　　　**Full Name:**

WITNESS CONSENT FORM FOR PARTICIPATION IN A RESEARCH STUDY

(For cases in which the participant is unable to read and/or write)

The impartial witness must be identified and must be a person not involved with the research team.

Study Title: *The Phenomenon of Centenarians in Ourense*

I, ..., as an impartial witness, confirm that in my presence:

- The Participant Information Sheet for the above-mentioned study was read aloud to ...., who was given the opportunity to ask any questions about the study.
- The participant understood that their participation is voluntary and that they may withdraw from the study at any time, without needing to provide an explanation and without this affecting their medical care.
- The participant agreed to the use of their data under the conditions detailed in the Participant Information Sheet.
- The participant freely gave their consent to participate in this study.

At the end of the study, the participant agrees that their data will be:

- **Deleted**
- **Retained in anonymized form for future use in other research**

Signed: The Witness **Signed:** The Researcher Requesting Consent

Full Name: **Full Name:**

Date: **Date:**

CONSENT FORM FOR LEGAL REPRESENTATIVE FOR PARTICIPATION IN A RESEARCH STUDY

Study Title: *The Phenomenon of Centenarians in Ourense – Quantitative and Qualitative Study*

I, **____**, legal representative of

- Have read the Participant Information Sheet for the above-mentioned study that was provided to me. I was able to speak with .... and ask all questions I had about the study.
- I understand that participation is voluntary and that the participant may withdraw from the study at any time, without needing to provide an explanation and without this affecting their medical care.
- I agree to the use of the participant's data under the conditions detailed in the Participant Information Sheet.
- I freely give my consent for the participant to take part in this study.

At the end of this study, I agree that their data will be:

- **Deleted**
- **Retained in anonymized form for future use in other research**

Signed: Legal Representative **Signed:** Researcher Requesting Consent

Full Name: **Full Name:**

Date: **Date:**

Section S4: Questionnaire

INSTRUCTIONS

1. Use the interviewee’s usual language.
2. Whenever possible, responses should be contrasted with information from a knowledgeable informant (children or relatives familiar with the interviewee's habits). The questionnaire may also be completed exclusively by the informant in cases of cognitive impairment. In such cases, a telephone interview may be acceptable.
3. Begin each question in an open-ended format. After posing the question, the interviewer should ask the respondent to explain or elaborate. If necessary, the question may then be closed with the multiple-choice options provided.

DATA COLLECTION

- Age:
- Gender: Male / Female
- Residence: Rural / Semi-urban / Urban
- Number of children:
- Marital status:
- Place of birth:
- Life trajectory (places of residence during different life stages):

Family Background

- At what age did your parents pass away (excluding preventable or unnatural deaths)?
  (Father, mother)
- At what age did your siblings pass away (excluding preventable or unnatural deaths)?
- Are there any other centenarians in your family?

Socioeconomic Status

- **Early-life socioeconomic level**
    - What was your parents' occupation? Skilled / Unskilled labor
    - How would you describe your family's financial situation during childhood? Better / Similar / Worse than neighbors
- **Adult socioeconomic level**
    - How would you describe your financial situation during adulthood? Better / Similar / Worse than average

---

Health

- **Self-perceived health**
    - In general, how do you perceive your health now? And throughout your life? (Open-ended)
    - Would you say your health is: Good / Fair / Poor
    - Do you experience daily pain? A lot / Quite a bit / Little / None

---

- **Medication** *(ask caregiver)*
    - Polypharmacy: How many different medications do you take daily? More than 5 / Less than 5
    - Since when have you been taking more than two medications? Before age 85 / Between 85–100 / After 100
    - What chronic medications do you take? (check all that apply): Antidiabetics, Anticoagulants, Antiplatelets, Antihypertensives, Lipid-lowering drugs, Antineoplastics, Anti-inflammatories, Antidepressants, Anxiolytics, Antipsychotics, Other
    - Have you ever participated in a clinical trial? Yes / No

---

Diet

- How would you describe your diet over your lifetime (childhood, adolescence, adulthood)? (Open-ended)
- Was your food intake generally large or small? A lot / Quite a bit / Little / None
- How many meals per day did you typically eat?
- What did you usually have for breakfast?
- Did you eat a lot of bread or potatoes (carbohydrates)? A lot / Quite a bit / Little / None
- Did you eat a lot of meat? A lot / Quite a bit / Little / None
    - What types of meat?
- Did you eat a lot of fish? A lot / Quite a bit / Little / None
    - What types of fish?
- Did you eat a lot of fruit? A lot / Quite a bit / Little / None

    - Favorite fruit and others: grapes, blueberries, blackberries
- Did you eat many legumes (fava beans, chickpeas, lentils)? A lot / Quite a bit / Little / None
- Did you eat a lot of vegetables? A lot / Quite a bit / Little / None
    - What kinds (kale, cabbage, chard, spinach, turnip greens, collard greens, green beans)?
- Did you eat many garden vegetables (tomato, carrot, pepper, lettuce, beet, garlic, onion, others)?
- Were your vegetables homegrown or purchased?
- Did you consume many sweets, sugar, honey, chocolate?
- Did you eat with a lot of or little salt?
- Did you drink a lot of milk? A lot / Quite a bit / Little / None
- Did you eat a lot of cheese or cottage cheese?
- Did you drink wine? A lot / Quite a bit / Little / None — Red or white?
- Did you drink coffee?
- Did you consume spirits, such as aguardiente or coffee liqueur?
- Was there anything you refused to eat? (Specify)
- How would you describe your weight for most of your life? Overweight / Normal / Thin

---

Water

- What do you think about the importance of drinking water?
- Were you someone who drank a lot of water? A lot / Quite a bit / Little / None
- What type of water did you drink? Bottled / Delivered / Well or spring
- How much water did you drink daily? (Estimate in glasses or liters)
- What kind of water did you use to irrigate your garden?

Thermalism

- What are your thoughts on thermal or medicinal waters?
- Did you visit thermal baths or spa resorts? A lot / Quite a bit / Little / None
- How many times per year did you visit?
- How many days each visit?
- Which thermal spas did you visit? (Specify)

Physical Activity

- Were you someone who moved a lot? Why?
- Did your work involve intense physical activity (that made you breathless or raised your heart rate)?
- Did you practice any sport in your free time?

- Did you engage in daily physical activity (walking, gardening, sports)?

Sleep

- What advice would you give for sleeping well? (Open-ended)
- **Sleep quality:** Were you satisfied with your sleep?
- Did you have trouble falling or staying asleep, or wake up too early?
- Did you use sleeping pills?
- **Sleep quantity:** Were you a heavy sleeper?
- How many hours did you typically sleep? More than 7 / 5–7 / Less than 5
- Did you take naps?

Intellectual Activity

- **Reading:** Did you read regularly? Why?
- **Education:** What level of education did you reach? Primary / Secondary / Higher education
- **Cognitive ability:** Were you a good student?

Perceived Stress

- Did you feel in control of your life?
- Were you frequently nervous? Why?
- **Stressful life events:** What were the most difficult moments in your life? (e.g., loss of spouse, child, imprisonment)
- **Financial stress:** Did you have enough money to live comfortably?

Occupational Dimension

- **Industriousness:** Were you someone who worked hard? Why?
- Were you ever an emigrant? Yes / No — How many years? Where? What was your experience?
- Did you often feel bored, or were you always occupied? High / Moderate / Low activity

---

Social Support

- **Emotional support:** Did you have someone to talk to when needed?
- **Material support:** Did you have help when sick or unable to complete tasks?

- **Social/recreational:** Did you have someone to enjoy leisure time with?
- **Affective support:** Did you have someone to love and who made you feel loved?
- Did someone worry about you?

Personal Attitudes & Resilience

- **Stability:** Were you easily angered or irritated?
- Were you a worrier?
- Were you a cheerful person?
- Were you calm and relaxed?
- **Friendliness:** Did you get along well with people?
- Did you have enemies?
- Were you kind in your speech?
- Were you emotionally sensitive?
- **Conscientiousness:** Were you orderly?
- Were you methodical or habit-driven?
- Were you reliable in your tasks?
- **Curiosity:** Were you full of ideas?
- Did you do a variety of activities?
- Did you start new ventures?
- Did you stay informed about current events?
- **Extraversion:** Did you enjoy being around people?
- Were you talkative?
- Did you have many friends?
- Did you enjoy parties and social events?
- **Resilience:** Did you bounce back easily from adversity?
- Were you able to adapt to changes easily?
- Did you move on quickly after failure?
- Did you tend to see the bright side of things?

Spirituality

Inner Peace
- Did you usually feel at peace with yourself? A lot / Quite a bit / Little / None

Religious Practice
- Were (and are) you a very religious person (e.g., someone who frequently attended mass)? A lot / Quite a bit / Little / None

Religious Belief
- Were (and are) you someone who spoke to or made requests of God? A lot / Quite a bit / Little / None

Satisfaction

- **Marital satisfaction:** Are you satisfied with how your marriage turned out? A lot / Quite a bit / Little / None
- **Sexual activity:** Was sex a significant part of your life? A lot / Quite a bit / Little / None
- **Life satisfaction:** Are you satisfied with your life up to now? A lot / Quite a bit / Little / None
    - Do you feel you achieved the important things in life? A lot / Quite a bit / Little / None
    - If you could live your life again, how much would you change? A lot / Quite a bit / Little / None

Self-perception of Longevity

- **Advice:** What do you think is the reason you have lived so long? Please share three (or more) tips for living a long life.

Section S5

Table S3: Pfeiffer Test

The Pfeiffer Test, also known as the SPMSQ (Short Portable Mental Status Questionnaire), is a 10-question assessment designed to evaluate cognitive impairment in older adults. It measures short- and long-term memory, orientation (to time, place, and person), knowledge of everyday facts, and the ability to perform simple arithmetic calculations.

No.	Question	Correct response (example)
1	What is today's date?	(day, month, and year)
2	What day of the week is it?	(e.g., Monday)
3	What is the name of this place?	(name of the hospital, care home, or residence)
4	What is your telephone number?	(acceptable to answer "I don't have one")
5	How old are you?	(exact age)
6	When were you born?	(day, month, and year)
7	Who is the current Prime Minister?	(correct name)
8	Who was the previous Prime Minister?	(correct name)
9	What is your mother's maiden name? (or maiden surname if female)	(correct surname)
10	Count backwards by threes from 20 (20, 17, 14, 11, 8…)	(correct sequence)

Scoring:

- Each incorrect answer = **1 point**.
- Up to **3 errors** are allowed in individuals with limited formal education.

Number of errors	Interpretation (standard educational background)
0 – 2 errors	Intact cognitive function
3 – 4 errors	Mild cognitive impairment
5 – 7 errors	Moderate cognitive impairment
8 – 10 errors	Severe cognitive impairment

Table S4: Barthel Test

The Barthel Index measures a person's ability to perform ten basic activities of daily living (ADLs), allowing for the assessment of their level of functional independence and need for assistance. These activities include eating, bathing, dressing, grooming, bowel and bladder control, using the toilet, transferring (e.g., from bed to chair), walking, and going up and down stairs. In summary, the Barthel Index assesses:

- Functional independence: The ability to carry out everyday activities without help.
- Level of dependency: The extent to which assistance is needed to perform these tasks.
- Disability progression: It can be used to monitor the development or recovery of a person's disability over time.

Activity	Score Options	Score
**1. Feeding**	Independent	10
	Needs help (cutting, spreading butter, etc.)	5
	Dependent	0
**2. Bathing**	Independent	5
	Dependent	0
**3. Grooming**	Independent (face/hair/teeth/shaving – implements provided)	5
	Dependent	0
**4. Dressing**	Independent	10
	Needs help	5
	Dependent	0
**5. Bowels**	Continent	10
	Occasional accident	5
	Incontinent	0
**6. Bladder**	Continent	10
	Occasional accident or catheter	5
	Incontinent	0
**7. Toilet use**	Independent	10
	Needs some help	5
	Dependent	0
**8. Transfers (bed to chair and back)**	Independent	15
	Major help (one or two people, physical aid)	10
	Dependent	0
**9. Mobility (on level surfaces)**	Independent	15
	Wheelchair independent	5
	Immobile or dependent	0
**10. Stairs**	Independent up and down	10
	Needs help	5
	Unable	0

Total Score	Interpretation
0–20	Total dependence
21–60	Severe dependence
61–90	Moderate dependence
91–99	Slight dependence
100	Independent

Figure S1: Assessment of physical activity, sleep, and other health habits of centenarians.

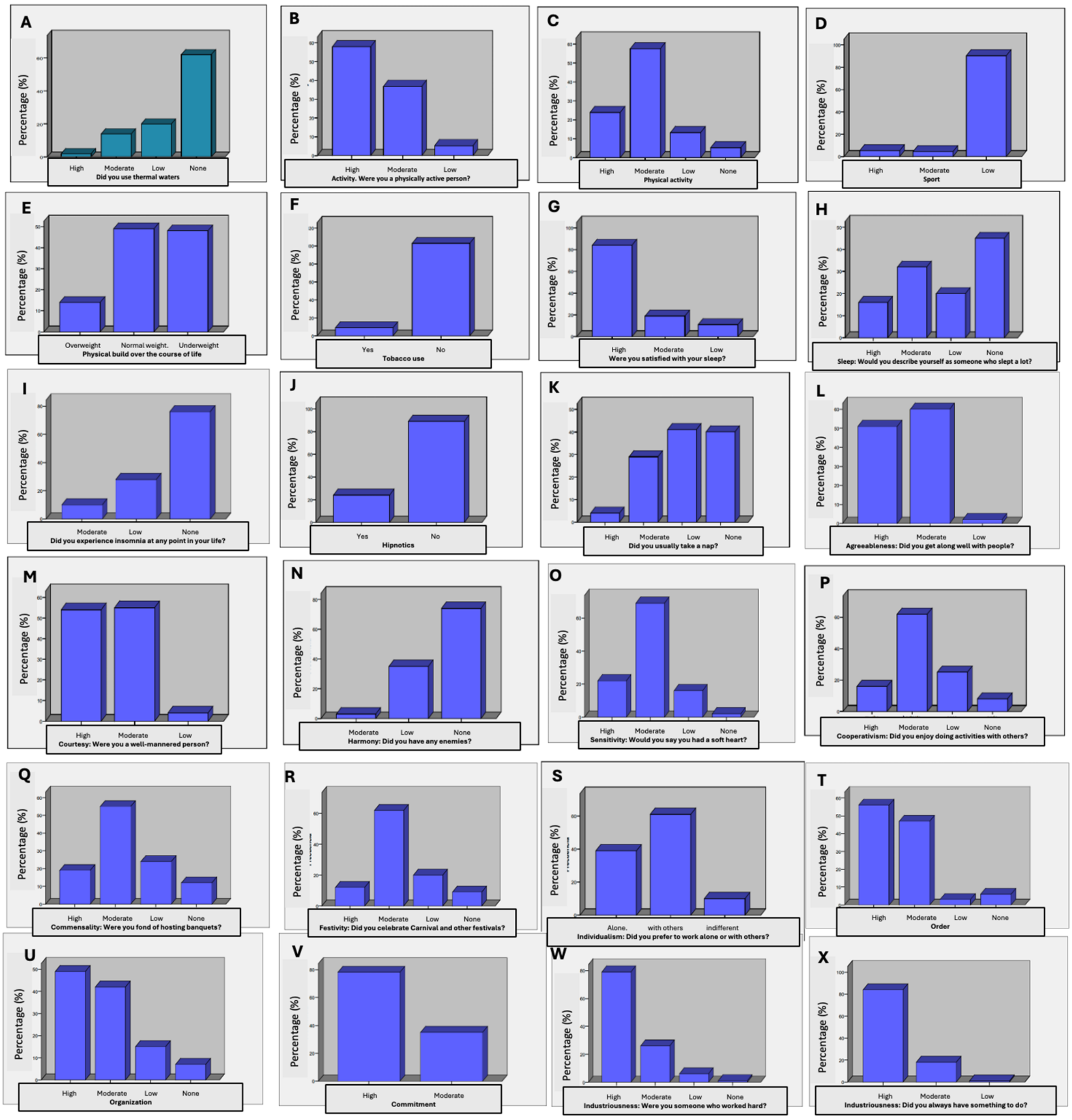

Figure S2: Assessment of emotional factors in centenarians.

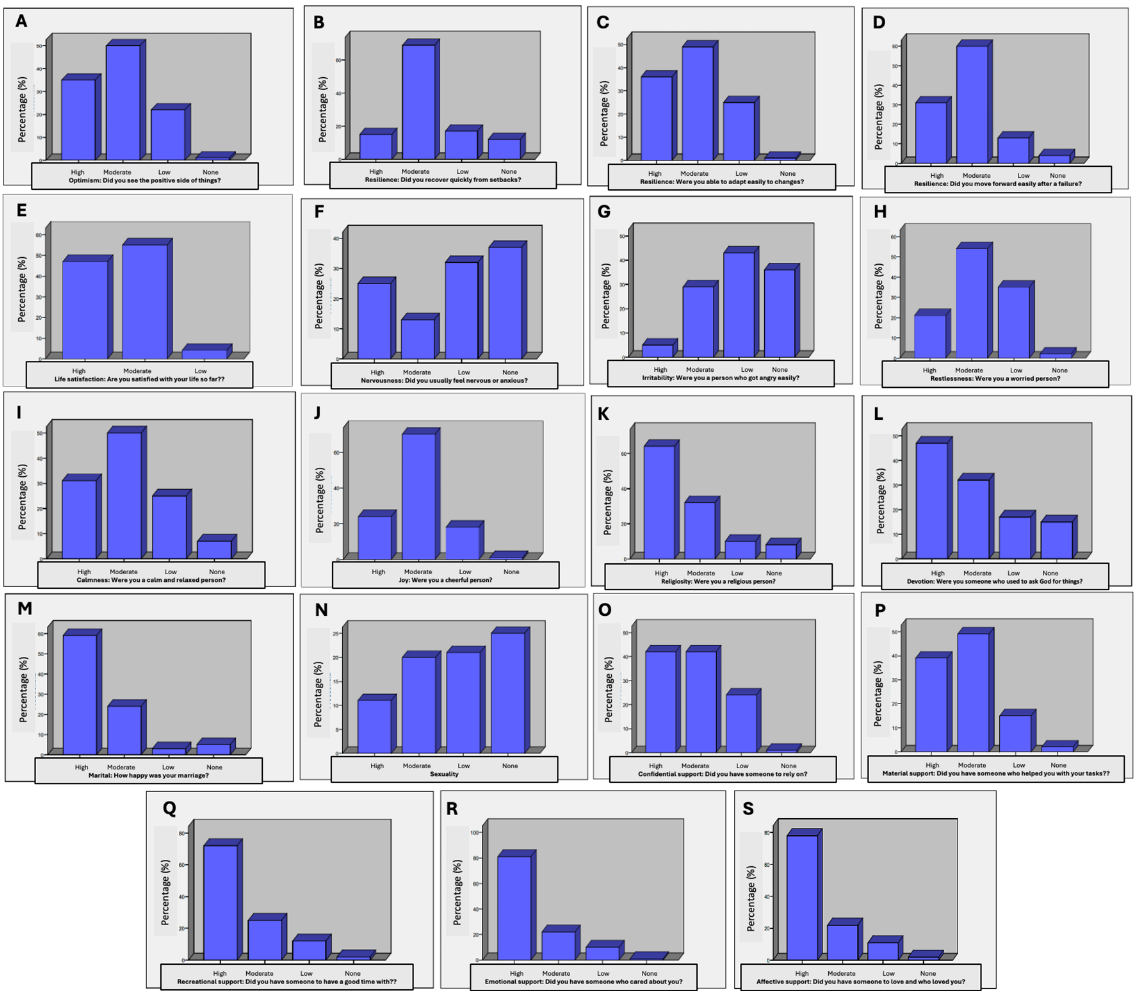

Figure S3: Study of factors influencing dementia and other aspects affecting longevity.

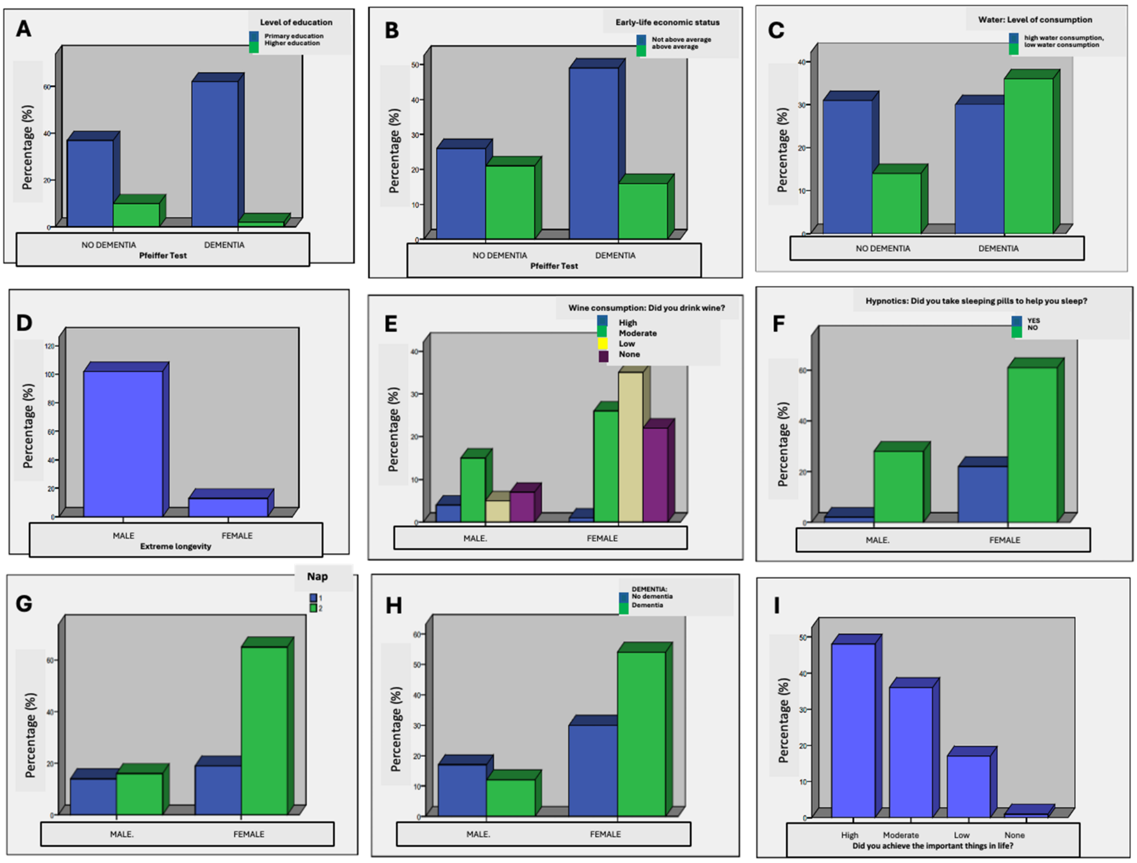

Supplement: Supplementary file 1 [file nutrients-17-02231-s001.zip › nutrients-3703732-supplementary.pdf]
